# Vaccine-Induced CD8^+^ T Cell Responses in Children: A Review of Age-Specific Molecular Determinants Contributing to Antigen Cross-Presentation

**DOI:** 10.3389/fimmu.2020.607977

**Published:** 2020-12-23

**Authors:** Elisabeth M. S. Beijnen, Simon D. van Haren

**Affiliations:** ^1^ Utrecht Institute for Pharmaceutical Sciences (UIPS), Faculty of Science, Utrecht University, Utrecht, Netherlands; ^2^ Precision Vaccines Program, Division of Infectious Diseases, Boston Children’s Hospital, Boston, MA, United States; ^3^ Department of Pediatrics, Harvard Medical School, Boston, MA, United States

**Keywords:** vaccine, vulnerable population, CD8, children, cross-presentation

## Abstract

Infections are most common and most severe at the extremes of age, the young and the elderly. Vaccination can be a key approach to enhance immunogenicity and protection against pathogens in these vulnerable populations, who have a functionally distinct immune system compared to other age groups. More than 50% of the vaccine market is for pediatric use, yet to date vaccine development is often empiric and not tailored to molecular distinctions in innate and adaptive immune activation in early life. With modern vaccine development shifting from whole-cell based vaccines to subunit vaccines also comes the need for formulations that can elicit a CD8^+^ T cell response when needed, for example, by promoting antigen cross-presentation. While our group and others have identified many cellular and molecular determinants of successful activation of antigen-presenting cells, B cells and CD4^+^ T cells in early life, much less is known about the ontogeny of CD8^+^ T cell induction. In this review, we summarize the literature pertaining to the frequency and phenotype of newborn and infant CD8^+^ T cells, and any evidence of induction of CD8^+^ T cells by currently licensed pediatric vaccine formulations. In addition, we review the molecular determinants of antigen cross-presentation on MHC I and successful CD8^+^ T cell induction and discuss potential distinctions that can be made in children. Finally, we discuss recent advances in development of novel adjuvants and provide future directions for basic and translational research in this area.

## Introduction

British physician Edward Jenner marked the beginning of vaccinology when he developed the world’s first vaccine for smallpox in 1796 (1). His invention relied foremostly on the awareness that dairymaids infected with cowpox were immune to outbreaks of smallpox. The next breakthrough occurred in 1880, when the French chemist Louis Pasteur discovered the principle of attenuation (2). Five years later, Pasteur produced the first laboratory-developed vaccine which tremendously increased the speed of vaccine development.

Most historically developed successful vaccines use weakened or inactivated pathogens. Examples of such vaccines are whole cell pertussis vaccine, which led to large and rapid reductions in pertussis deaths in the United States after its introduction in 1914, (3, 4), or the inactivated polio vaccine which has successfully eradicated poliomyelitis (**Table 1**) (24). More recently, however, technological developments have shifted vaccine development toward the production of formulations that do not contain live material, such as nucleic acid vaccines and subunit vaccines. Subunit vaccines are comprised of purified protein or polysaccharide antigens, often combined with adjuvants, immune potentiators that are capable of stimulating the immune system (24). The first successful example is the hepatitis B subunit vaccine, derived from the hepatitis B surface antigen (HBsAg) (**Table 1**). The development of subunit vaccines has led to improved safety profiles, inclusion of immunostimulants to drive specific types of immune responses, and the opportunity for vaccine component optimization. However, the more defined composition of subunit vaccines can lead to challenges as well, as seen in the case of pertussis vaccination. Replacement of whole-cell pertussis vaccine (wP) by acellular pertussis vaccine (aP), a subunit vaccine, has led to a resurgence of pertussis due to ‘waning immunity’ (25, 26). The efficacy of subunit vaccines often relies on appropriate type and magnitude of immune activation by adjuvants. As the majority of the global vaccine market is for pediatric use, there is an unmet need to critically review the mechanism of action of these adjuvants in a pediatric setting. Studies on adjuvant mechanism of action in early life from our group and others thus far have focused predominantly on the induction of cytokines, antibodies and CD4^+^ T cells (27–34), but much less is known about the activation of CD8^+^ T cells in early life, and the ability of vaccine formulations or adjuvants to induce these.

**Table 1:** Vaccines that are licensed in human newborns and infants in the United States.

Licensed pediatric vaccine	Vaccine type	Antigen(s)	Type(s) of adjuvant (5–7)	Evidence of CD8^+^ T cell mediated immunity in control of infection
Hepatitis A virus (HAV)	Inactivated	Inactivated hepatitis A virus (strain HM175)	Virosomes, aluminum hydroxide	Yes (limited data) (8–10)
Trivalent inactivated influenza vaccine (TIV)	Inactivated	Hemagglutinin	Virosomes, MF59, AS03	No (8, 11–14)
Inactivated poliovirus (IPV)	Inactivated	D antigen	None	Yes (15, 16)
Rotavirus (RV)	Live attenuated	Spike protein	No adjuvant used	Unclear (17, 18)
Bacillus Calmette-Guérin (BCG)	Live attenuated	Antigen 85	None	Yes (9, 10, 19)
Measles, mumps, rubella	Live-attenuated	Trivalent antigen	None	Measles: yes (18) Mumps: no (20)
Varicella (VAR)	Live attenuated	Varicella virus live	None	Yes (11–14, 21)
Live attenuated Influenza vaccine (LAIV)	Live attenuated	Hemagglutinin	None	Yes (11–13, 22)
Hepatitis B virus (HBV)	Subunit	HBsAg	Virosomes, AS04	Yes (16, 23)
Diphtheria, Tetanus & acellular Pertussis (DTaP)	Toxoid, subunit	Tetanus toxoid, diphtheria toxoid, detoxified pertussis toxin	Aluminum hydroxide	Yes (acellular Pertussis) (15, 19)
Pneumococcal conjugate (PCV)	Conjugate	Pneumococcal polysaccharides conjugated to a nontoxic form of diphtheria toxin CRM197	Aluminum phosphate	No (limited data) (21, 23)
Haemophilus influenzae type b (Hib)	Polysaccharide conjugate	Polysaccharide conjugated to Hib bacterium	None or with aluminum hydroxide	?

In vaccine development, quantitative correlates of protection are often determined by quantification of serum antibody levels or neutralizing ability (35, 36). In antiviral vaccine development, however, absolute correlates of protection are not always defined, and relief of symptoms due to eradication of viral disease is a good indicator of vaccine success. Viruses are intracellular pathogens and use the host cell’s machinery for internalization, translation of viral proteins and viral genome replication (37). Upon viral infection, a cell can use endogenously generated cytosolic viral proteins for antigen presentation *via* major histocompatibility complex (MHC) I molecules on its surface. MHC class I molecules can be found on the cell surface of all nucleated cells (38). CD8^+^ T cells recognize short peptides derived from antigenic proteins presented by these molecules and, hence, play a critical role in the control and elimination of viral infections. MHC class II molecules are expressed on antigen presenting cells (APCs), such as dendritic cells (DCs) (39). CD4^+^ T cells, which recognize peptides presented by MHC class II molecules, promote antibody production which is in many cases sufficient for protection against viruses. While other APCs such as B cells and macrophages are important during different stages of T cell activation, this review will focus on DCs and their role in the instruction of naive T cells.

Activated CD8^+^ T cells can induce apoptotic death of virus-infected cells by the production of Tumor necrosis factor-alpha (TNF-α), Interferon-gamma (IFN-γ) and the release of cytotoxic molecules containing granzymes, perforins, and granulysin (40, 41). These effector functions directly contribute to pathogen clearance. In childhood, when the highest risk for infection exists, protective antibodies decline rapidly after primary vaccination (42). Newborns and infants are highly susceptible to viral infectious diseases and impaired CD8^+^ T cell responses may lead to progressive or even fatal infection. For example, there is evidence that SARS-CoV-2 virus can infect children (43–47) and can sometimes have severe consequences, such as multisystem inflammatory syndrome in children (MIS-C) (45, 48). SARS-CoV-2-specific CD8^+^ T cells are detectable in infected and convalescent individuals, and potentially correlate with disease outcome (49–52). Vaccine induced CD8^+^ T cell priming may therefore improve the efficacy of immunization in infants against viral pathogens (53, 54).

Nucleic acid-based vaccines and subunit vaccines do not contain a live vector and are therefore generally more safe than inactivated and live attenuated vaccines. However, the high purity of the components can make these vaccines less immunogenic and hence potentially less effective (42), if not adjuvanted properly. Nucleic acid vaccines rely on incorporation of the genetic material into the host antigen-presenting cell genome, potentially resulting in endogenous transcription of viral proteins and therefore effective presentation on MHC class I. Subunit vaccines are composed of only antigenic viral proteins or carbohydrates and therefore the step of genome incorporation into the host is removed. As a consequence, the antigen will not gain access to the cytosol, which is known to be a critical step for MHC class I presentation and subsequent CD8^+^ T cell activation. In general, nucleic acid vaccines are therefore more effective in eliciting CD8^+^ T cell responses (55–57). To improve immunogenicity of subunit vaccines, adjuvants can be added to the formulation. Adjuvants promoting CD8-mediated immunity are therefore a key element for developing effective subunit vaccines against viruses. This can be accomplished by the process of cross-presentation, which enables MHC class I presentation of viral proteins, taken up from extracellular sources. Evidence of adjuvant-induced cross-presentation has been described, often including a proposed mechanism of action (58–68). However, there is to date no published data describing whether and how adjuvants induce cross-presentation in early life. In this review, we address the key concept of how adjuvants can activate CD8^+^ T cell responses and discuss their ability to regulate key molecular pathways relating to antigen cross-presentation in early life (46). Understanding the functionality of CD8^+^ T cells in early life and how they can be effectively induced by adjuvants directly informs the development of subunit vaccines for pediatric use.

## Changes in Frequency of CD8^+^ T Cells With Age

An important parameter for the induction of an effective antiviral response is that there is a sufficient number of CD8^+^ T cells available to extirpate virus-infected cells. T cell precursors arise from hematopoietic stem cells (HSCs), which are composed of two main cell populations: Sca-1^-^ lymphoid-biased stem cells, and Sca-1^+^ myeloid-biased stem cells. Lymphocytopoiesis in infants is distinguished by the robust production of T cells, due to a relatively high number of lymphoid-biased HSCs. However, these cells decline with increasing age and as a consequence, the ratio of HSCs in adults shifts toward more myeloid biased HSCs. These cells are less efficient in creating common lymphoid progenitors with high proliferative capacity compared to their counterparts, which directly contributes to the reduction in naive T cell generation in the aged population (69). In addition to a greater influx of HSCs with lymphoid potential into the thymus in children, mouse studies have shown greater efficacy in thymopoiesis in early life (70, 71), resulting in a higher frequency of naive CD8^+^ T cells in the periphery (72, 73). This latter observation is also seen in humans, as both the frequencies of recent thymic emigrants (RTEs) (74, 75) and of naive CD8^+^ T cells (76) decreases with age. Other factors that affect the functioning of HSCs with increasing age are oxidative stress and reduced telomerase activity, which cause the naive CD8^+^ T cell compartment to shrink gradually (77, 78).

In support of the foregoing, experimental data indicate that young infants exhibit higher frequencies of CD8^+^ T cells compared to their adult counterparts. Young adults carry roughly 10^11^ CD8^+^ T cells (79). Absolute values of neonatal CD8^+^ T cells in human are absent, but limiting dilution studies have shown that the precursor frequency of CD8^+^ cytolytic T cells in neonates is comparable to that in adults (80). In fact, Thome et al. observed that infants (0 – 2 years) express significant higher percentages of CD8^+^ naive T cells compared to young adults (15–25 years) in circulation, lymphoid and mucosal tissues (81).

## Phenotypic and Functional Differences of CD8^+^ T Cells Between Age Groups

### Phenotypic Differences

In addition to distinctions in frequency of total as well as naive CD8^+^ T cells with age, the expression of certain cell surface receptors can differ between age groups as well, potentially affecting vaccine response to infection or to vaccination (**Table 2**). The main distinctions observed in receptor expression relate to the maturity or activation status of the CD8^+^ T cells. In accordance with findings that newborns and infants have higher levels of naive CD8^+^ T cells, a higher percentage of CD8^+^ T cells express CD28. CD28 serves as a co-stimulator for T-cell activation and survival and is expressed on all naive T cells in newborns (87). In elderly cells, CD28 expression is diminished and sometimes even lost (**Table 2**). This likely contributes to impaired immune responses in elderly. Nevertheless, CD28^-^ T cells express higher levels of effector molecules such as perforin and granzyme B and therefore show improved cytotoxicity (92). This supports the difference in cytotoxicity level between adults and infants, as will be discussed in the next paragraph.

**Table 2:** Non-exhaustive list of CD8+ T cell marker levels in different age groups.

Phenotypic (CD8^+^) T cell marker	Level of neonatal versus adult/elderly expression in T cells	Reference
αβ-TCR	Similar	(82, 83)
CD3	Similar	(83–85)
CD5	Similar	(83, 86)
CD8	Similar	(83)
CD28	Lower expression in adults; 40-50% of the elderly (age 80 and above) lack CD28 expression	(87)
CD38	Higher expression in neonates	(83, 88)
KIR	Higher expression in adults and elderly	(89)
CD45RA^lo^CD45RO^lo^	Expressed by neonates, rare or absent in adult T cells	(83, 90)
CD300a	Higher expression in adults	(91)

Another activation marker, CD38 is also more frequently expressed on neonatal or infant CD8^+^ T cells compared to adult CD8^+^ T cells (**Table 2**). CD38 is expressed early in ontogeny and is suggested to play an important role during T cell activation (93). In the context of human immunodeficiency virus (HIV) infection, high proportions of CD38^+^CD8^+^ T cells are associated with virologic worsening (88). However, there are studies that have observed opposite findings in children (94, 95). Thus, the significance of CD38 distinctions in the CD8 compartment with age still remains unclear and needs to be further examined in different age groups.

At baseline, children age 6–15 and age 16–17 have similar levels of central memory CD8^+^ T cells compared to adults, but significantly less effector memory CD8^+^ T cells (96). Upon activation with staphylococcal enterotoxin B (SEB), the increase in expression of activation marker CD69 was significantly reduced in these cells, in particular in the 6–15 age group.

Effector CD8^+^ T cells can be distinguished by Killer cell Immunoglobulin-like Receptors (KIR) expression. KIR^+^ cells are estimated to represent approximately 5% of the CD8^+^ T cells in adults and can increase up to 30% in elderly individuals (89). In contrast, roughly 1.67% of CD8^+^ T cells express KIRs in cord blood (97). CD8^+^ T cells acquire KIRs when differentiating into effector molecules (98). This confirms that neonates have more naive T cells than their adult counterparts. The biological functions of KIRs on T cells remain poorly understood although it has been shown that these receptors enhance the efficiency of HLA class I-mediated CD8^+^ T cell responses (99) and therefore could positively influence the outcome of viral infections.

Upon activation, CD8^+^ T cells can introduce the expression of inhibitory molecules aiming to prevent an immoderate immune response. One of these receptors is CD300a, a transmembrane protein with immunoreceptor tyrosine-based inhibitory motifs (ITIMs) capable of conduct inhibitory signaling (100). In a comparative study exploring the CD300a expression on human neonatal versus adult immune cells, significant differences in presence of CD300 receptors on CD8^+^ T cells derived from cord blood and adult blood were observed. The research group showed that naive and memory CD8^+^ T cells from cord blood exhibited significant lower levels of CD300a when compared to adult T cells (91).

In summary, expression profiles of activation CD8^+^ T cell markers correlate with age, displaying more activated T cells when older, due to repeated antigen exposure.

### Functional Differences

Neonatal and adult lymphocytes exhibit differential expression of genes involved in T cell receptor (TCR) signaling. Notably, with regards to the neonatal TCR pool, it has been proposed that neonatal T cells may be less dependent on TCR recognition than their adult counterparts (101). TCRs are integral membrane proteins, which control T cell activation through recognition of specific peptides presented by MHC molecules (102). Neonatal T cells exhibit a less diverse TCR repertoire than adult T cells due to a lag in expression of the enzyme terminal deoxynucleotidyl transferase (TdT) (101). TdT is responsible for adding nontemplated (N) nucleotides in V, D, and J gene segments of TCRs (103) and hence plays an important role in diversifying these receptors. Diversification in TCR signaling is of essence, because a larger pool of different TCRs increases the possibility of recognizing all kinds of peptide antigens. Interestingly, the diverse TCR repertoire of adult CD8^+^ T cells diminishes with increasing age, which contributes to increased susceptibility to viral infections (104).

Upon TCR stimulation, newborn CD4^+^ T cells favor the secretion of IL-8 but less IFN-γ secreting T-helper 1 cells are observed as compared to adult CD4^+^ T cells (101). This is a result of impaired production of type-1-polarizing cytokines by neonatal DCs in response to stimulation through Toll-like Receptors (29, 105). This also affects the CD8 compartment, resulting in CD8^+^ T cells with a more type-2 phenotype (Tc2), which can exacerbate allergy-type reactions in asthma or infection with respiratory syncytial virus (RSV) (106–109).Thus, the immune response generated by neonatal T cells is more of an innate nature, whereas adults produce cytokines that are typically associated with adaptive immune responses. Furthermore, neonatal T cells are less likely to secrete multiple cytokines simultaneously (110). In other words, neonatal T cells are less polyfunctional, which could subsequently lead to less potent T cell responses (96). In a recent study on HIV-1 responses by CD8^+^ T cells, the results showed that HIV-1 specific adult CD8^+^ T cells with high frequencies of CD300a were more polyfunctional (111). These observations are in line with the difference in CD300a expression levels between adults and neonates, as described in the previous paragraph.

Galindo-Albarrán et al. have observed that neonatal T lymphocytes are less cytotoxic than adult CD8^+^ T cells due to lower expression of IFN-γ, a signature molecule for activating the cytolytic pathway (112). Furthermore, they showed that certain enhancers of cytotoxic genes were only expressed in adults and that neonatal CD8^+^ T cells express only low numbers of granzyme producing cells. Interestingly, expression levels of granzyme B by neonatal NK cells are found to be similar or even higher than adult NK cells (113). Therefore, it could be postulated that neonatal NK cells are being deployed as a compensation mechanism for having CD8^+^ T cells bearing low cytotoxicity.

In elderly, differentiation of CD8^+^ T cells into effector molecules has shown to be impaired in response to infection due to reduced expression of important cytokines, such as IFN-γ, TNF-α, granzyme B, and IL-2 (114). Another functional decline of the immune response in the elderly is suggested to originate from down regulation of certain genes in CD8^+^ T cells which affect a variety of stages of gene transcription, such as transcription initiation, elongation, RNA stabilization, and protein translation and translocation (115). Certainly, more studies are required to fully understand the primary causes of the impaired gene expression that occurs in CD8^+^ T cells in the older population and their functional consequences.

Another significant discrepancy between newborn and adult CD8^+^ T cells is that neonatal cytotoxic T cells have higher proliferative rates than adult naive CD8^+^ T cells and, subsequently, differentiate more rapidly into effector cells (116). As a consequence, an imbalance in effector and memory CD8^+^ T cell formation emerges in neonatal cells, with a shift toward more CD8^+^ T cell effector cells. Thus, newborn cells are less capable of creating immunological memory which has direct implications for creating adaptive immune responses after re-infection. It has been suggested that differences in microRNA (miRNA) expression profiles are accountable for these findings. miRNAs are non-coding mRNA molecules that modulate different aspects of immune responses, such as T cell differentiation. Wissink et al. observed that age-dependent changes in miR-29 and miR-130 in human CD8^+^ T cells may contribute to the diminished development of neonatal memory cells (117). Further research is required to support this hypothesis.

Age-related changes in CD8+ T cell frequency and proliferation rate may also be influenced by the presence of homeostatic cytokines, such as IL-7. IL-7 plays a central role in maintaining T cell homeostasis and serves as a key factor in the proliferation and survival of naive T cells (118, 119). During thymic development, stromal and epithelial cells in the thymus produce IL-7 to promote CD8^+^ T cell differentiation in the thymus (120). Thymic production declines with age and, as a consequence, IL-7 levels may decrease during the aging process (121). This could negatively affect CD8+ T cell expansion in response to vaccination and potentially result in failure of immunization.

Fms-like tyrosine kinase 3 ligand (FL) also functions as an important regulator of hematopoiesis and is widely distributed in both murine and human tissues (122). FL has an important role in regulating immunity, due to its capacity to stimulate the expansion of DCs (123). Its receptor, Fms-like tyrosine kinase 3 (FLT3), is mostly expressed by immature hematopoietic cells and shows similar expression patterns in newborn and adult mice (122). To our knowledge, however, no differences in FL levels among age groups have been reported.

## Ontogeny of Vaccine Induced CD8^+^ T Cell Responses

**Table 1** lists the commercially available vaccines for pediatric and adult use in the United States. The majority of the live attenuated or inactivated vaccines do induce protective CD8^+^ T cell mediated immunity, providing empiric evidence that there is at least no impairment in MHC class I loading or CD8^+^ T cell functionality in early life. Empiric evidence of protective CD8^+^ T cell mediated immunity induced by protein-based vaccines is much less substantial (**Table 1**). There are different mechanisms to create CD8^+^ T cell responses after immunization. Modern vaccines may use viral vectors or nucleic acids as a vaccine delivery system (124). These delivery systems are attractive for vaccine therapy because of their capability to provoke potent and sustained CD8^+^ T cell responses (57, 125). However, the kinetics of nucleic acid delivery and expression of the antigen by APCs likely makes adjuvantation very challenging. Enhancement of the immune response to nucleic acid-based vaccines can be achieved by inclusion of plasmids that encode cytokines, costimulatory receptors, or Toll-like receptor (TLR) ligands (126, 127).The ability to instruct appropriate (often Th1-mediated) CD4^+^ T cell responses in newborns and infants is impaired (128, 129) and requires adjuvantation with select molecules or combinations that have shown the ability to overcome this impairment (27, 29, 130).

**Figure 1:**
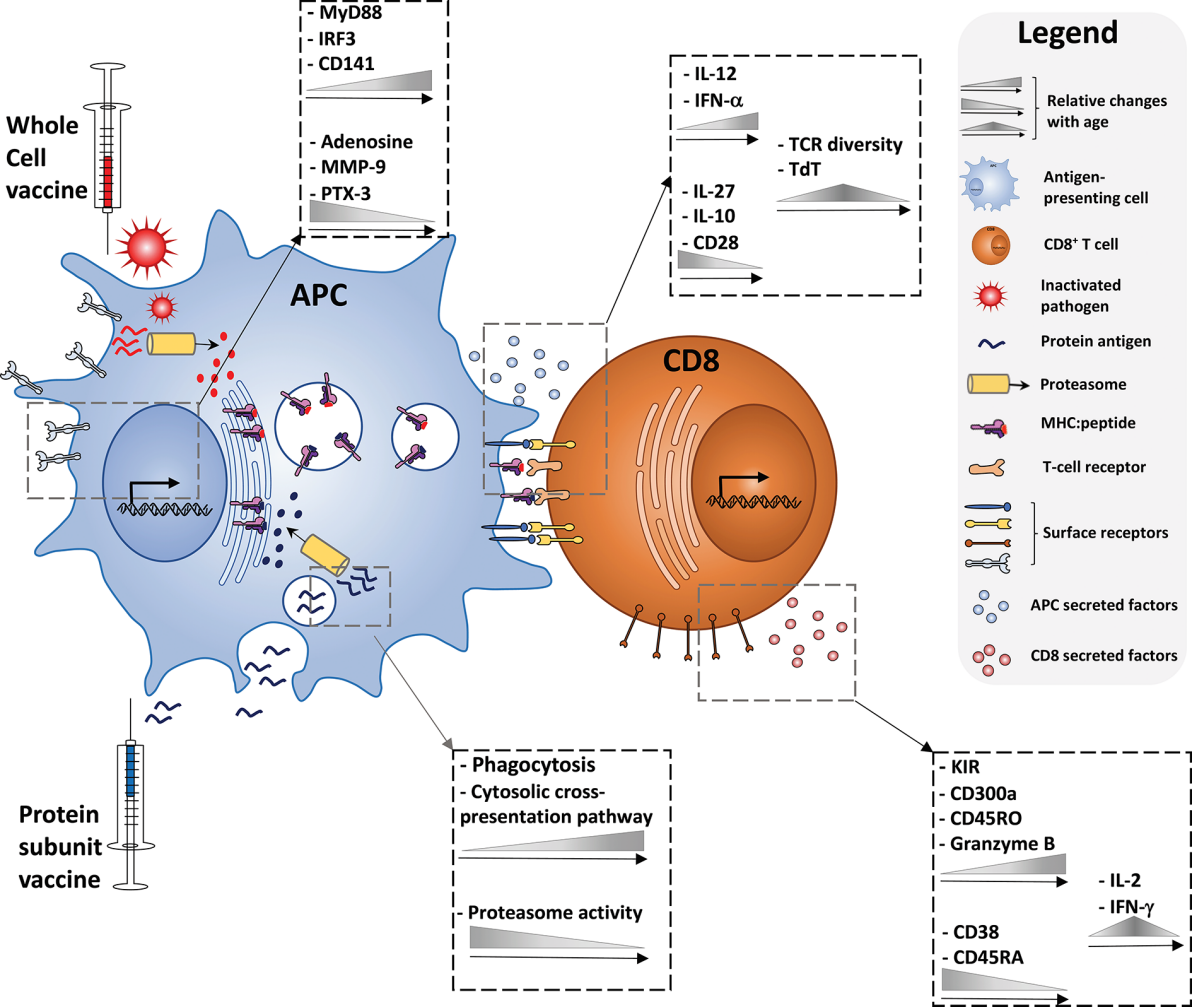
Immune ontogeny of a vaccine-induced CD8^+^ T cell response. Starting with recognition of vaccine antigens by APCs (left), up to the effector phenotype of a vaccine-induced CD8^+^ T cell (right), relative changes with age of key cytokines, receptors, and biological processes discussed in this review are depicted.

An alternative method for inducing CD8^+^ T cell responses is through the mechanism of cross-presentation in which MHC class I molecules present exogenous peptides to naive CD8^+^ T cells. This is in contrast to classical MHC class I presentation, in which a foreign peptide will be displayed after it has arrived into the cytosol of the cell inter alia after the cell has been infected. Antigen cross-presentation has been studied for decades, since its discovery in 1976 (131, 132), but there are still many aspects of this concept which are controversial and not fully understood. However, it is clear that there are different subcellular pathways involved in cross-presentation, each consisting of crucial steps for MHC class I presentation. In order to evaluate the potential of adjuvants to induce cross-presentation in children, it is important to summarize the components and mechanisms of cross-presentation to the extent that they are currently known and understood. **Figure 1** provides an illustrated summary of the different components, cytokines, receptors, and biological processes contributing to successful vaccine-induced CD8+ T cell activation discussed in this review, and the extent to which changes with age have been observed.

## Human Dendritic Cell Subsets and Cross-Presentation

Dendritic cells are a class of bone-marrow-derived cells which can be found in blood, tissues and lymphoid organs. They are referred to as ‘professional’ APCs because of their unique ability to bridge the innate and adaptive immune system *via* the presentation of antigens to naive T cells. In human, dendritic cells are divided between two major lineages: conventional DCs (sometimes called myeloid DCs) and non-classical DCs. Based on their phenotypic and functional characteristics, these populations are further compartmentalized into several subtypes (**Table 3**). Each subset is specialized to react to particular pathogens and to interact with specific T cell subsets. In this manner, the immune system can act upon a broad spectrum of several pathogens and danger signals.

**Table 3:** DC subsets functions and distinctive markers.

Subset	Cross-presents?	Function(s)	Distinctive markers
cDC1	Yes (133)	cross-presentation (134) Necrotic cells uptake (135) Alloactivation (136) Promote Th1 polarization (137)	CD141, XCRI, CLEC9A, CADM1
cDC2-A	Yes (133, 138–140)	Promote Th1/Th17 polarization (141)	CD11c, CD1c, CD32^+^
cDC2-B	Yes (133, 138, 142)	Promote Th1/Th17 polarization (141)	CD36, CD1c, CD163
Dermal cDC1	?	?	CD141, CD11c
Dermal Langerin^-^ cDC2	?	?	CD1a, CD11c
Dermal Langerin^+^ cDC2	?	Promote Th1 polarization, inhibit Th17 cell differentiation (murine model) (143)	Langerin, CD1a, CD11c
pDC	Yes (144–146)	Promote antiviral immune responses (type I IFN production) (147, 148) Th2 polarization (149) Pathogenic functions in autoimmunity (148) Tolerogenic functions: can induce suppressive responses by inducing Tregs through IDO expression (147)	CD123, BDCA2, BDCA4
CD14^+^ DC	No (134, 144, 150, 151)	Tolerogenic functions: Treg induction (152) Th2 polarization (153)	CD209 (154)
SLAN DC	?	Produce Th17-programming cytokines and induce Th17/Th1 cells (155) Promote proliferation, cytotoxicity and IFN-α production by NK cells (156)	SLAN, CD16
IDEC	?	Th1 polarization, recruitment of inflammatory cells, amplification of allergic-inflammatory reactions (149)	CD1a, CD11c
Tip DC	?	Might be important for immunoglobulin A production (157, 158) Th1 polarization *in vitro* (159) Can stimulate the differentiation and activation of Th17 cells, may participate in tumor rejection (158)	iNos, TNF

Current vaccination strategies take into account the functional specialization of different DC subsets. For example, both the CD1c^+^ subset (also known as cDC2 DCs) and the CD141^+^ subset (also known as cDC1 DCs) have potent capacity to induce T cell responses. Where cDC2 cells are predominantly inducers of CD4+ T cell responses, cDC1 cells are uniquely able to cross-present exogenous antigens on MHC I. Interestingly, neonatal cDC1^+^ DCs reach adult-like levels by mid-gestation (160), and therefore, this subpopulation may be a desirable tool for vaccine development to empower antiviral immunity in early life.

In literature, the chemokine receptor XCR1 is presented as a universal surface marker on cross-presenting DCs (161) in mice as well as humans. This marker is also present on cDC1^+^ DCs and, therefore, it is thought that XCRI^+^ DCs are crucial in creating successful adaptive immune responses against viruses (162). In addition, pDCs, which do not express XCR1, are considered to cross-present in humans (144–146). However, the exact role of pDCs in cross-presentation remains controversial (163, 164).

Full-term newborns and adult pDCs display similar frequencies in whole blood, although subset composition between these age groups may differ (165). However, Zhang et al. observed that these differences do not affect the potency of neonatal antiviral responses (166). In contrast, pDCs from preterm newborns have shown an immature morphology and an impaired capacity to produce IFN-α (165).

It should be noted that it is difficult to determine the functional distinctions with age of DC subsets in humans. To study the characteristics of DC types *in vitro*, studies are mainly carried out with moDCs. For neonates, moDCs are generated from umbilical cord blood. One of the limitations thereof is the presence of maternal factors in the content of the blood, which may influence the characterization of neonatal DCs (167). However, due to the convenience of this method, moDCs are the main subset for studying the phenotype and function of DCs.

## The Mechanism of Antigen Cross-Presentation

### The Role of Endocytosis: Soluble Versus Particulate Antigens

Cross-presentation of soluble and particulate antigens is regulated by distinct methods of internalization. Particulate antigens are selectively internalized by APCs through phagocytosis. Subsequently, the antigen can be presented through both MHC class I and II molecules, a time-dependent process in which the NADPH oxidase 2 (NOX2) plays a crucial part. This enzyme is found in professional phagocytes and DCs and contributes to the alkalization of phagosomes by ROS production. NOX2 is recruited to phagosomes with the help of Rab27a and Rac2 (168, 169). Thus, NOX2 prevents phagosome acidification and, consequently, abolishes lysosomal antigen degradation which then allows for cross-presentation (170). This means that when ROS production ceases and the phagosomal pH gets more acidic, the particulate antigen will be preferentially loaded onto MHC class II molecules (171).

In contrast to cell-associated antigens, soluble antigens intended for cross-presentation are internalized by endocytic receptors. Burgdorf et al. describe two different endocytic compartments for antigen processing: early endosomes and lysosomes (172). If a soluble antigen is routed into a lysosome, classical MHC II presentation will take place, whereas antigens in endosomes are targeted for presentation on MHC class I molecules. Depending on the type of endocytic receptor the antigen interacts with upon internalization, the antigen will be sorted into one of the compartments, a process taking place at the plasma membrane (172, 173). Receptors used by DCs to take up extracellular antigens and route these into endosomal compartments include the C-type lectin receptors CLEC9a, DC-SIGN, Mannose Receptor, and DEC-205 (172, 174, 175). Furthermore, molecular chaperones such as heat shock proteins (HSP) can also bind exogenous antigens for MHC class I presentation, through the scavenger receptors LOX1 and SCARF1 (176).

Interestingly, in newborns, monocytes and neutrophils exhibit a reduced ability to bind and ingest particles. This impairment is transient as neonatal phagocytic ability has shown to reach adult-like levels after a few days after birth (177). There are many factors that potentially account for this phenomenon. For example, the chemotaxis of cord blood phagocytes is decreased and Fcγ receptor expression is diminished in early life. Furthermore, newborns show reduced numbers of neutrophils with phagocytic capacity and display poor complement activity (178). Notably, it has been observed that preterm infants with low numbers of neutrophils contain higher phagocytic ability compared to term infants (179).

### The Cytosolic Pathway: Critical Steps in Antigen Cross-Presentation

The cytosolic pathway is characterized by translocation of internalized soluble or particulate antigens to the cytoplasm where they go through degradation by large protein complexes, referred to as proteasomes (59). The way antigens translocate across the endocytic membrane into the cytoplasm is still debated. It has been suggested that proteins require an unfolding step before translocation (180). However, experimental studies observed enzymatically active proteins in cytosolic extracts, proposing that these proteins do not get unfolded (150).

A common theory is that antigens are transported into the cytosol by sec61, a member of the endoplasmic reticulum associated degradation (ERAD) machinery (181). However, there are papers that have suggested that cytosol export can be independent of sec61 (182, 183). Sec61 has additional functions relating to protein transport across ER and plasma membranes, making it challenging to explore its exact contribution to antigen cross-presentation.

As mentioned previously, low phagosomal pH prevents cross-presentation of particulate antigens. However, it should be emphasized that a slightly acidic environment in the phagosome is required for transportation into the cytosol (180). Particulate antigens can form aggregates and therefore should be processed before transportation. This means that the phagosomal pH should be strictly regulated to prevent antigens from excessive degradation but still be able to deliver them into the cytosol (184, 185).

The involvement of the soluble N-ethylmaleimide-sensitive fusion protein attachment protein receptor (SNARE) sec22b, located in the ER-Golgi intermediate compartment (ERGIC), as a mediator of antigen export to the cytosol has been described in many papers. However, in recent literature, the role of sec22b in cross-presentation has been questioned (186, 187). Overall, whether sec22b is critical for antigen cross-presentation remains under investigation.

After antigens undergo protein degradation in the cytosol, the proteasome-generated peptides subsequently follow two possible routes: the antigens are transported back into the endosome [1] or into the ER lumen [2], of which the latter only applies to cell-associated antigens (188). The import of peptide fragments into the ER is suggested to occur *via* the transporter associated with antigen processing (TAP). This protein was also found in antigen-containing lysosomes, supporting the hypothesis that peptide loading could also occur inside the lysosomal compartment (189). Indeed, it has been observed that selective TAP deficiency in endosomes strongly impaired the ability for cross-presentation (190). However, TAP-independent pathways also have been described (191, 192). It has been observed that the majority of cytosolic peptides that are being processed TAP-independently are derived from C terminal ends of proteins or N-terminal signal sequences (193). Many proteases are thought to be involved in this process. It should be noted, however, that direct evidence for ER peptide loading is missing. This means that the exact site of peptide loading has not been clarified yet.

If peptides are routed back into the endosomal compartment, efficient cross-presentation requires the translocation of ER proteins to the endosome. ER protein trafficking takes place with the help of sec22b and syntaxin 4, a transmembrane SNARE member present on phagosomes. In this manner, ERGIC molecules such as sec61 and TAP are recruited to phagosomes and endosomes (184). Furthermore, the ER-associated aminopeptidase 1 (ERAP) and the endosomal insulin-responsive aminopeptidase (IRAP) are recruited to trim the antigens to obtain the right size for efficient MHC class I complexing (194).

With regards to newborns, Kollman et al. studied the efficacy of cross-presentation in murine neonatal dendritic cells using soluble ovalbumin (OVA) (195). Their results showed a clear reduction in neonatal MHC class I presentation of the soluble antigen, while antigen uptake in neonates and adults were similar. As OVA cross-presentation is dependent on the cytosolic pathway (180, 196), this evidence implies that the cytosolic pathway may be impaired in early life.

### The Vacuolar Pathway

Unlike the cytosolic pathway, internalized antigens that follow the vacuolar route do not reach the cytosol. Instead, the antigens are thought to be both degraded and loaded onto MHC class I molecules inside the phagosome or endosome. In literature, TAP (in)dependency is mainly used as a determining criterion to distinguish between the cytosolic and vacuolar pathway. However, as mentioned, the cytosolic pathway could also occur without the involvement of the TAP transporter. Besides, research has indicated that cross-presentation of long peptides through the vacuolar pathway can be TAP dependent (197). Therefore, it seems that this distinction does no longer holds ground.

It has been postulated that active proteases, such as the cysteine protease cathepsin S, can enter the endosome or phagosome to process internalized antigens into smaller peptides (198). However, it has been argued that the variety of hydrolases within phagosomes is too harsh for the production of 8-16 amino acid peptides, required for MHC class I loading (199). This argument might not provide sufficient grounds against the fact that there are approximately 15 degradative peptidases and over 50 acid hydrolases localized in the cytosol available for antigen processing *via* the cytosolic pathway (200).

It is not known whether the vacuolar pathway in newborns and children is fully competent. Human neonatal APCs show distinct features in terms of expression of costimulatory molecules, and therefore it has been proposed that these cells require a higher level of activation than their adult counterparts in order to create similar CD8^+^ T cell responses (201). Considering these data, once a human neonatal APC is activated, it could still be entirely competent to induce an adaptive effector response. In support of this notion, Gold et al. found no defect in human neonatal DCs to process and present particulate antigen and concluded that cross-presentation is fully functional in human newborn DCs. However, as previously described, Kollman et al. observed otherwise (195). It could be proposed that differences between these studies might be due to dissimilarities in engagement of the vacuolar pathway. Another possibility is that these different findings are partially caused by the type of antigenic form used in the experiments. It is known that particulate and soluble antigens have distinct immunologic properties. For example, particulation ensures targeted delivery of antigens to APCs in a more concentrated form and, subsequently, results into an adjuvant effect (202). Furthermore, the antigen within the particle is exhibited in multiple copies, leading to more robust and persisting cellular responses. In light of the foregoing, it could be possible that the intrinsic properties of particulate antigens offset the mediocre costimulatory support displayed by human neonatal APCs.

There are many other facets of cross-presentation still to be elucidated. For example, it is unknown whether the role of the TAP-transporter and sec22b are age-dependent. Furthermore, animal experiments suggest that proteasome function might be elevated in early life and decline with age (203, 204). In brief, there is an unmet need to conduct research on the MHC I pathway in early life and the age-dependent aspects of this process.

### Important Cytokines

In order to obtain a functional cytotoxic T cell response, the sole presence of antigens is not adequate. Instead, pro-inflammatory cytokines and costimulatory molecules are required to create an inflammatory environment that will activate naive CD8^+^ T cells. Several cytokine receptors, such as IL-12R and the type I interferon receptor, are essential to activate key transcription factors that support cellular immunity. However, as mentioned earlier, the neonatal immune system demonstrates a characteristic impairment in the production of Th1 polarizing cytokines, such as IFN-α and IL-12p70, which imposes challenges on creating robust and sustained CD8^+^ T cell responses (29, 205–213). Although cell-intrinsic components contribute to this distinct functionality of newborn DCs, elevated plasma levels of extrinsic factors such as IL-10, adenosine, MMP-9, and PTX-3 (214–216) can also play a role.

IFN-α is a type I interferon (IFN), which is predominantly produced by pDCs *in vivo*. When PRRs such as TLRs and cytosolic RIG-I-like receptors recognize viral proteins, early type I IFN production is initiated. Type I IFNs play a major role in antiviral immunity, as they are capable of upregulating MHC and costimulatory molecules on DCs (205). Besides, through direct CD8^+^ T cell contact, type I IFNs significantly improve clonal expansion of CD8^+^ T cells *in vivo* (206). It is well known that type I IFN levels, such as IFN-α, correlate with age. Indeed, newborns infected with RSV show a significant decline in IFN-α production compared to adults (207). It has been postulated that pDC functionality is impaired in newborns and, therefore, shows poor IFN-α induction (208).

Production levels of IL-12 are notably lower in newborns and infants compared to adults (209). Recent work showed that TCR/IL-12 stimulation can enhance expression of genes in newborns that are associated with T cell functions, including cytotoxicity genes and cell signaling genes (210). The ability of newborn dendritic cells to produce IL-12p70 in response to TLR agonists proposedly can be overcome by combined stimulation through TLR4 and Dectin-1 (213). In this study, however, dendritic cells were generated from cord blood monocytes (moDCs) in the presence bovine serum before activation. We have previously demonstrated that the ability of newborn moDCs to produce IL-12p70 is highly reduced by soluble factors present in cord plasma, and impaired Th1 induction was instead overcome independently of IL-12p70 production (29).

Another member of the IL-12 family, IL-27, is a cytokine which consists of both inflammatory and immunosuppressive capabilities. One of its functions is to promote the survival and differentiation of CD8^+^ T cells, thereby contributing to their effector functions (211). IL-27 secretion by dendritic cells is highest in childhood, while adults’ levels are low (212). Interestingly, Il-27 helps drive T helper 1 (Th1) cell differentiation, while newborns are impaired in inducing this type of immune response. The pleiotropic nature of IL-27 could make it difficult to determine its contribution to the impaired Th1 response observed in newborns.

## Adjuvant-Induced Cross-Presentation

Several studies have described potential mechanisms of cross-presentation induced by clinically relevant adjuvants, such as aluminum, saponin and toll like receptor agonists. The next paragraphs elaborate on the molecular pathways of these adjuvants. However, very little is known about these mechanisms in newborns, and therefore, more research is required in order to comment on potential age-dependent differences between these adjuvants.

### Aluminum-Based Adjuvants

Insoluble aluminum (alum) salts are the most broadly used classical adjuvants in human vaccines (217). Alum is known for its ability to provoke strong T helper 2 (Th2) responses but does not typically enhance CD8^+^ T cell-mediated immunity.

Alum salts are particulate adjuvants comprised of crystalline structures, which are thought to be central to their adjuvanticity. It has been shown that alum induces the production of uric acid (218). Uric acid can precipitate into crystals of monosodium urate (MSU), which can be phagocytosed by APCs. Phagocytosis of particulate matter, such as alum or MSU, can trigger disruption of the phagosomal membrane, resulting into the activation of the NOD-like receptor protein 3 (NLP3) inflammasome. In addition, alum has also been shown to induce cell death, leading into the release of danger signals like DNA and uric acid. These components are also able to activate the NLP3 inflammasome (218). However, the role of NLP3 in cross-presentation is likely to be limited since NLPR3 is a transcriptional regulator of Th2 differentiation (219). In support of this notion, alum has shown to be capable of inducing a CD8^+^ T cell response without the involvement of the inflammasome (217).

Interestingly, alum-based nanoparticles in combination with the TLR ligand cpG showed enhanced cross-presentation by DCs (220). With the use of endocytic pathway inhibitors, it was observed that the scavenger receptor A was responsible for internalization of the alum-polymer particles. The nanoparticles were both found in the lysosome and cytosol, indicating lysosomal escape. In addition, both brefeldin A, which inhibits ER transport to the Golgi apparatus, and MG-132, a proteasome inhibitor, reduced alum-induced cross-presentation in DCs. A potential reason for this enhancement in response could be the involvement of both the cytosolic and vacuolar pathway. This is, because it has been speculated that TLR ligands potentially use the vacuolar pathway (58), while alum-based adjuvants seem to follow the cytosolic pathway. Activating both routes of cross-presentation may enhance MHC class I restricted presentation and, thus, promote CD8^+^ T cell mediated immunity. There are many other factors that could play a role, such as particle size and manufacturing conditions.

### Saponin-Based Adjuvants

Saponins are triterpene plant glycosides that exhibit different biological and pharmacological properties. There are several saponins that can stimulate the immune system which has led to significant interest in their potential as vaccine adjuvants (221). The most extensively investigated saponin adjuvant is QS-21, a purified fraction from the soap bark tree (*Quillaja Saponaria*) (222).

The molecular composition of QS-21 revealed that its aldehyde group is key in inducing cellular immunity. This is, because it was observed that after reduction of the aldehyde moiety into a secondary amine, adjuvanticity was lost (223). The immune stimulating role of aldehyde-containing adjuvants has been previously described, such as in case of lipidated tucaresol (224). QS-21 is thought to provide a costimulatory signal to the T cell through imine formation from its aldehyde and the primary amine on the T cell, most likely CD2 (221). However, the aldehyde group is not likely to play a role in cross-presentation because tucaresol is not able to induce CD8^+^ T cell immunity by itself. Furthermore, there are also existing triterpene saponins that lack imine-forming structural groups but still induce cytotoxic T cells against exogenous antigens (223).

Saponin-antigen complexes enter the APC by endocytosis in a cholesterol-dependent way (221). Den Brok et al. proposed that, once the antigen-saponin complex is engulfed by the membrane, MHC class I presentation is induced through lipid body formation (225). As previously described, LBs potentially facilitate antigen export to the cytosol and would therefore play an important role in inducing CD8^+^ T cell responses. LB formation destabilizes the membrane and, therefore, allows the antigen to escape the endosome early (221). Thus, antigen translocation into the cytosol occurs in a proteasome-independent matter. Indeed, saponin-induced cross-presentation was not compromised by different NAPDH oxidases and several ROS scavengers.

Surprisingly, pharmacological inhibition of LB induction did not reduce antigen export to the cytosol. However, pharmacological and genetic interreference with lipid body formation did abrogate saponin-induced cross-presentation. Thus, LBs might contribute to saponin-mediated CD8^+^ T cell immunity in a different yet undefined matter.

### TLR-Based Adjuvants

DCs express different subtypes of TLRs on their surface. TLRs recognize various PAMPs and therefore play an important role in immunosurveillance. Increasing evidence shows that TLR signaling is involved in multiple steps in cross-presentation. It was found that TLR activation controls several aspects of phagocytosis like internalization and phagosome maturation. For example, TLR signals accelerate both phagocytosis and phagolysosomal fusion (226). DC activation status plays a critical role in this process. Indeed, it was shown that activation of DCs with TLR3 and TLR4 ligands significantly reduced the uptake and subsequent cross-presentation of particulate antigen compared with immature DCs (227). This phenomenon was not observed with TLR2 and TLR7 ligands. Another potential explanation for this difference is that TLR3 and TLR4 signaling require Trif as essential adapter, whereas the other TLRs operate Trif independent (228).

TLRs may also contribute to cross-presentation *via* MHC I enrichment, a process which is suggested to occur in a phagosome-autonomous way (59). Gupta et al. observed that TLR4 stimulation in murine BMDCs enhanced the recruitment of MHC class I molecules to phagosomes (229). In their work, they showed that these molecules were not derived from the ERGIC machinery, since recruitment of ERGIC components to phagosomes happened in a TLR-independent matter. This also suggests that TLRs are not involved in TAP recruitment, as proposed in literature (190). Instead, they suggested that MHC I molecules are recruited from the endosomal recycling compartment (ERC), regulated by the activity of rab11a. TLRs would manage this process through TLR-MyD88-IKK2-dependent phosphorylation of phagosomal SNAP-23.

Cross-presentation may be further enhanced through TLR mediated antigen export. Antigen transport from the phagosome to the cytosol was increased after TLR4 stimulation with LPS (227). This would suggest that TLR adjuvanticity favors the cytosolic pathway. However, this would not explain the previous described enhancement in MHC class I molecules in the phagosome, which suggests phagosomal loading instead of ER loading. Furthermore, TLRs accelerate phagosome maturation in the first hours after antigen uptake (230). Phagosome maturation in DCs allows antigens to be processed for antigen presentation. In this way, antigen degradation would not include the proteasome and, therefore, it could be argued that TLR ligands follow the vacuolar pathway. However, evidence points to the contrary, as many papers observed that the cytosolic pathway is ruling in TLR-mediated cross-presentation (68, 231, 232). Very little is known about the underlying molecular mechanism of adjuvant-induced cross-presentation in newborns. However, the type and magnitude of CD4 T cell activation by licensed adjuvants often differs, due to distinct signaling requirements in newborn antigen-presenting cells (233–237). To induce cross-presentation in neonates, TLR-adjuvants are interesting candidates for adjuvant application. TLR expression and downstream signaling have been well studied in newborns and although age distinctions have been observed, specific TLR agonists or combinations have been identified that can induce adult-like levels of pro-inflammatory cytokines such as type I IFNs and Il-12, which are important for cross-presentation and are generally not highly produced in newborn cells. Furthermore, TLR ligands appear to induce a similar degree of polyfunctionality compared to adults (110). However, IRF3 activation by TLR3 and TLR4 is reduced in newborns (238). This process is Trif-dependent and, as described in the previous chapter, TLR3 and TLR4 ligands showed reduced antigen uptake and cross-presentation, indicating that adjuvants stimulating these receptors will not induce cross-presentation in newborns as effectively.

Most TLR signaling is dependent on the adaptor protein myeloid differentiation primary response 88 (MyD88). It has been suggested that MyD88 functioning in neonatal DCs is impaired (239). As described above, MHC class I upregulation may take place in a MyD88 dependent way and, therefore, it could be postulated that TLR-mediated MHC I enrichment in newborns is reduced, possibly resulting in impaired cross-presentation. However, it has been shown that newborn cells can increase MyD88 mRNA expression after bacterial infection (240), and potent nuclear translocation of NF-kB can be achieved using TLR7/8 agonists rather than TLR3 or TLR4 agonists. Whether this would also happen upon viral infection is unknown. Even though alum-adjuvants are probably less suitable candidates in early life, because of their propensity to be Th2 skewing, combinations of alum with TLR adjuvants have shown promise, as described above.

## Concluding Remarks and Future Directions

This paper highlights key differences between the neonatal, infant, and adult immune system and aims to underline that our understanding of vaccine mediated CD8^+^ induction in early life requires further investigation.

Most commercially available vaccines for pediatric use consist of attenuated or inactivated pathogens. While these vaccines are mostly competent in stimulating CD8^+^ T cell immunity, modern vaccine development is shifting toward subunit and nucleic acid vaccines and, consequently, has imposed major challenges on inducing adequate cellular immunity. Therefore, subunit vaccines often depend on immune activation by adjuvants. Little is known about CD8^+^ T cell induction by adjuvants, for example, *via* cross-presentation, in newborns and infants. Adding to the complexity, in early life, many aspects of the immune system correlate with age. Even though neonates and infants have enough naive CD8^+^ T cells to create a robust antiviral response, they exhibit several functional differences compared to adults that may have direct implications for their ability to cross-present antigens. As a result, their CD8^+^ T cells have reduced cytotoxicity and are biased toward type 2 immunity. And neonatal APCs receive weak costimulatory stimulation. Altogether, this means that a vaccinated child will produce less pro-inflammatory cytokines important for cross-presentation, does not receive the same stimulation as an adult and shows poor CD8^+^ T cell effector properties. To overcome these hurdles in the pediatric population, adjuvants should be tailored to their distinct immune system.

Future research should examine whether cross-presentation mechanisms in neonates and infants are fully operational, and aim to identify adjuvants that can induce potent CD8^+^ T cell responses For example, using adjuvant combinations that employ both the vacuolar and cytosolic pathway or use different mechanisms for antigen export to the cytosol may enhance MHC class I presentation. Furthermore, antigen particulation can boost the adjuvant effect and outbalance poor neonatal APC costimulation. Besides, extra stimulation of cytokines such as Il-12 may enhance neonatal cytotoxicity and, thus, improve the antiviral response.

To date, however, it is unknown how adjuvants contribute to cross-presentation in neonates. For example, do TLR adjuvants also enhance antigen uptake and phagolysosomal fusion in newborns or is this an age-dependent process? Do adjuvants use similar cross-presentation pathways in newborns as they do in adults? Refining our understanding of adjuvant-induced CD8^+^ T cell immunity will further improve vaccine formulations in the pediatric setting and, hopefully, create more robust and sustained responses to protect this vulnerable population.

## Author Contributions

EB performed literature study. EB and SH wrote the manuscript. All authors contributed to the article and approved the submitted version.

## Funding

SH is supported by US National Institutes of Health (NIH)/National Institutes of Allergy and Infectious Diseases (NIAID) award Molecular Mechanisms of Combination Adjuvants (1U01AI124284-01). The Precision Vaccines Program is supported in part by the BCH Department of Pediatrics and the Chief Scientific Office.

## Conflict of Interest

The authors declare that the research was conducted in the absence of any commercial or financial relationships that could be construed as a potential conflict of interest.

## References

[1] Riedel S Edward Jenner and the History of Smallpox and Vaccination. Bayl Univ Med Cent Proc (2017) 18:21–5. 10.1080/08998280.2005.11928028 PMC1200696 16200144

[2] Smith KA Louis Pasteur, the Father of Immunology? Front Immunol (2012) 3:68. 10.3389/fimmu.2012.00068 22566949 PMC3342039

[3] Chow MYK Khandaker G McIntyre P Global Childhood Deaths From Pertussis: A Historical Review. Clin Infect Dis (2016) 63:S134–41. 10.1093/cid/ciw529 PMC5106618 27838665

[4] Klein NP Licensed pertussis vaccines in the United States. Hum Vacc Immunother (2014) 10:2684–90. 10.4161/hv.29576 PMC4975064 25483496

[5] Pasquale AD Preiss S Silva FTD Garçon N Vaccine Adjuvants: from 1920 to 2015 and Beyond. Nato Adv Sci Inst Se (2015) 3:320–43. 10.3390/vaccines3020320 PMC4494348 26343190

[6] Wilkins AL Kazmin D Napolitani G Clutterbuck EA Pulendran B Siegrist C-A AS03- and MF59-Adjuvanted Influenza Vaccines in Children. Front Immunol (2017) 8:1760. 10.3389/fimmu.2017.01760 29326687 PMC5733358

[7] Esposito S Meregalli E Daleno C Ghio L Tagliabue C Valzano A An open-label, randomized clinical trial assessing immunogenicity, safety and tolerability of pandemic influenza A/H1N1 MF59-adjuvanted vaccine administered sequentially or simultaneously with seasonal virosomal-adjuvanted influenza vaccine to paediatric kidney transplant recipients. Nephrol Dial Transpl (2010) 26:2018–24. 10.1093/ndt/gfq657 PMC7313880 20974645

[8] Walker CM Adaptive Immune Responses in Hepatitis A Virus and Hepatitis E Virus Infections. Csh Perspect Med (2018) 9:a033472. 10.1101/cshperspect.a033472 PMC6531370 29844218

[9] Sanchez-Schmitz G Levy O Development of Newborn and Infant Vaccines. Sci Transl Med (2011) 3:90ps27–7. 10.1126/scitranslmed.3001880 PMC4108897 21734174

[10] Murray RA Mansoor N Harbacheuski R Soler J Davids V Soares A Bacillus Calmette Guerin Vaccination of Human Newborns Induces a Specific, Functional CD8+ T Cell Response. J Immunol (2006) 177:5647–51. 10.4049/jimmunol.177.8.5647 17015753

[11] He X-S Holmes TH Zhang C Mahmood K Kemble GW Lewis DB Cellular Immune Responses in Children and Adults Receiving Inactivated or Live Attenuated Influenza Vaccines. J Virol (2006) 80:11756–66. 10.1128/jvi.01460-06 PMC1642596 16971435

[12] Schotsaert M Saelens X Leroux-Roels G Influenza vaccines: T-cell responses deserve more attention. Expert Rev Vaccines (2014) 11:949–62. 10.1586/erv.12.71 23002976

[13] Hoft DF Babusis E Worku S Spencer CT Lottenbach K Truscott SM Live and Inactivated Influenza Vaccines Induce Similar Humoral Responses, but Only Live Vaccines Induce Diverse T-Cell Responses in Young Children. J Infect Dis (2011) 204:845–53. 10.1093/infdis/jir436 PMC3156924 21846636

[14] Skibinski DAG Jones LA Zhu YO Xue LW Au B Lee B Induction of Human T-cell and Cytokine Responses Following Vaccination with a Novel Influenza Vaccine. Sci Rep-uk (2018) 8:18007. 10.1038/s41598-018-36703-7 PMC6301966 30573748

[15] Wahid R Cannon MJ Chow M Virus-Specific CD4+ and CD8+ Cytotoxic T-Cell Responses and Long-Term T-Cell Memory in Individuals Vaccinated against Polio. J Virol (2005) 79:5988–95. 10.1128/jvi.79.10.5988-5995.2005 PMC1091702 15857985

[16] Lumley SF McNaughton AL Klenerman P Lythgoe KA Matthews PC Hepatitis B Virus Adaptation to the CD8+ T Cell Response: Consequences for Host and Pathogen. Front Immunol (2018) 9:1561. 10.3389/fimmu.2018.01561 30061882 PMC6054973

[17] Desselberger U Huppertz H-I Immune responses to rotavirus infection and vaccination and associated correlates of protection. J Infect Dis (2011) 203:188–95. 10.1093/infdis/jiq031 PMC3071058 21288818

[18] Jaimes MC Rojas OL González AM Cajiao I Charpilienne A Pothier P Angel and J. Frequencies of Virus-Specific CD4 and CD8 T Lymphocytes Secreting Gamma Interferon after Acute Natural Rotavirus Infection in Children and Adults. J Virol (2002) 76:4741–9. 10.1128/JVI.76.10.4741-4749.2002 PMC136136 11967291

[19] Rieber N Graf A Hartl D Urschel S Belohradsky BH Liese J Acellular Pertussis Booster in Adolescents Induces Th1 and Memory CD8+ T Cell Immune Response. PloS One (2011) 6:e17271. 10.1371/journal.pone.0017271 21408149 PMC3050840

[20] de Wit J Emmelot ME Poelen MCM van Binnendijk RS van der Lee S van Baarle D Mumps infection but not childhood vaccination induces persistent polyfunctional CD8+ T-cell memory. J Allergy Clin Immun (2018) 141:1908–1911.e12. 10.1016/j.jaci.2017.11.047 29339261

[21] Frey CR Sharp MA Min AS Schmid DS Loparev V Arvin AM Identification of CD8 + T Cell Epitopes in the Immediate Early 62 Protein (IE62) of Varicella-Zoster Virus, and Evaluation of Frequency of CD8 + T Cell Response to IE62, by Use of IE62 Peptides after Varicella Vaccination. J Infect Dis (2003) 188:40–52. 10.1086/375828 12825169

[22] Mohn KG-I Zhou F Brokstad KA Sridhar S Cox R Live attenuated influenza vaccination boosts durable cross-reactive and protection-associated T-cells in children. J Infect Dis (2017) 215:jix165. 10.1093/infdis/jix165 PMC5461427 28368530

[23] Recher M Hirsiger JR Bigler MB Iff M Lemaître B Scherer K Immune system correlates of extensive limb swelling in response to conjugated pneumococcal vaccination. NPJ Vaccines (2018) 3:17. 10.1038/s41541-018-0059-3 29796310 PMC5959910

[24] Moyle PM Toth I Modern Subunit Vaccines: Development, Components, and Research Opportunities. Chemmedchem (2013) 8:360–76. 10.1002/cmdc.201200487 23316023

[25] Antunes R da S Babor M Carpenter C Khalil N Cortese M Mentzer AJ Th1/Th17 polarization persists following whole-cell pertussis vaccination despite repeated acellular boosters. J Clin Invest (2018) 128:3853–65. 10.1172/jci121309 PMC6118631 29920186

[26] Gu X-X Plotkin SA Edwards KM Sette A Mills KHG Levy O Waning Immunity and Microbial Vaccines—Workshop of the National Institute of Allergy and Infectious Diseases. Clin Vaccine Immunol (2017) 24:e00034–17. 10.1128/cvi.00034-17 PMC5498725 28490424

[27] Dowling DJ van HSD Scheid A Bergelson I Kim D Mancuso CJ TLR7/8 adjuvant overcomes newborn hyporesponsiveness to pneumococcal conjugate vaccine at birth. JCI insight (2017) 2:e91020. 10.1172/jci.insight.91020 28352660 PMC5360187

[28] van Haren SD Ganapathi L Bergelson I Dowling DJ Banks M Samuels RC In vitro cytokine induction by TLR-activating vaccine adjuvants in human blood varies by age and adjuvant. Cytokine (2016) 83:99–109. 10.1016/j.cyto.2016.04.001 27081760 PMC4906944

[29] van Haren SD Dowling DJ Foppen W Christensen D Andersen P Reed SG Age-Specific Adjuvant Synergy: Dual TLR7/8 and Mincle Activation of Human Newborn Dendritic Cells Enables Th1 Polarization. J Immunol (2016) 197:4413–24. 10.4049/jimmunol.1600282 PMC7386828 27793997

[30] Nguyen M Leuridan E Zhang T Wit D Willems F Damme P Acquisition of Adult-Like TLR4 and TLR9 Responses during the First Year of Life. PloS One (2010) 5:e10407. 10.1371/journal.pone.0010407 20442853 PMC2861003

[31] Zaghouani H Hoeman CM Adkins B Neonatal immunity: faulty T-helpers and the shortcomings of dendritic cells. Trends Immunol (2009) 30:585–91. 10.1016/j.it.2009.09.002 PMC2787701 19846341

[32] Lissner MM Thomas BJ Wee K Tong A-JJ Kollmann TR Smale ST Age-Related Gene Expression Differences in Monocytes from Human Neonates, Young Adults, and Older Adults. PloS One (2015) 10:e0132061. 10.1371/journal.pone.0132061 26147648 PMC4493075

[33] Lee AH Shannon CP Amenyogbe N Bennike TB Diray-Arce J Idoko OT Dynamic molecular changes during the first week of human life follow a robust developmental trajectory. Nat Commun (2019) 10:1092. 10.1038/s41467-019-08794-x 30862783 PMC6414553

[34] Reikie BA Adams RC Ruck CE Ho K Leligdowicz A Pillay S Ontogeny of Toll-Like Receptor Mediated Cytokine Responses of South African Infants throughout the First Year of Life. PloS One (2012) 7:e44763. 10.1371/journal.pone.0044763 23028609 PMC3441420

[35] Plotkin SA Correlates of Protection Induced by Vaccination▿. Clin Vaccine Immunol (2010) 17:1055–65. 10.1128/cvi.00131-10 PMC2897268 20463105

[36] Plotkin S Complex Correlates of Protection After Vaccination. Clin Infect Dis (2013) 56:1458–65. 10.1093/cid/cit048 23386629

[37] van Montfoort N van der Aa E Woltman AM Understanding MHC class I presentation of viral antigens by human dendritic cells as a basis for rational design of therapeutic vaccines. Front Immunol (2014) 5:182. 10.3389/fimmu.2014.00182 24795724 PMC4005948

[38] Hewitt EW The MHC class I antigen presentation pathway: strategies for viral immune evasion. Immunology (2003) 110:163–9. 10.1046/j.1365-2567.2003.01738.x PMC1783040 14511229

[39] Koszinowski UH Reddehase MJ Jonjic S The role of CD4 and CD8 T cells in viral infections. Curr Opin Immunol (1991) 3:471–5. 10.1016/0952-7915(91)90005-l 1684507

[40] Harty JT Tvinnereim AR White DW CD8+ T cell effector Mechanisms in Resistance to Infection. Annu Rev Immunol (2000) 18:275–308. 10.1146/annurev.immunol.18.1.275 10837060

[41] Schmidt ME Varga SM The CD8 T Cell Response to Respiratory Virus Infections. Front Immunol (2018) 9:678. 10.3389/fimmu.2018.00678 29686673 PMC5900024

[42] Castellino F Galli G Giudice GD Rappuoli R Generating memory with vaccination. Eur J Immunol (2009) 39:2100–5. 10.1002/eji.200939550 19637203

[43] Shekerdemian LS Mahmood NR Wolfe KK Riggs BJ Ross CE McKiernan CA Characteristics and Outcomes of Children With Coronavirus Disease 2019 (COVID-19) Infection Admitted to US and Canadian Pediatric Intensive Care Units. JAMA Pediatr (2020) 174(9):868–73. 10.1001/jamapediatrics.2020.1948 PMC7489842 32392288

[44] Zimmermann P Curtis N Coronavirus Infections in Children Including COVID-19: An Overview of the Epidemiology, Clinical Features, Diagnosis, Treatment and Prevention Options in Children. Pediatr Infect Dis J (2020) 39:355–68. 10.1097/inf.0000000000002660 PMC7158880 32310621

[45] Jiang L Tang K Levin M Irfan O Morris SK Wilson K COVID-19 and multisystem inflammatory syndrome in children and adolescents. Lancet Infect Dis (2020) 20(11):E276–88. 10.1016/s1473-3099(20)30651-4 PMC7431129 32818434

[46] Kelvin AA Halperin S COVID-19 in children: the link in the transmission chain. Lancet Infect Dis (2020) 20(6):P633–4. 10.1016/s1473-3099(20)30236-x PMC7156154 32220651

[47] Lu X Zhang L Du H Zhang J Li YY Qu J SARS-CoV-2 Infection in Children. New Engl J Med (2020) 382:1663–5. 10.1056/nejmc2005073 PMC7121177 32187458

[48] Saleem H Rahman J Aslam N Murtazaliev S Khan S Coronavirus Disease 2019 (COVID-19) in Children: Vulnerable or Spared? A Systematic Review. Cureus (2020) 12:e8207. 10.7759/cureus.8207 32577325 PMC7305578

[49] Peng Y Mentzer AJ Liu G Yao X Yin Z Dong D Broad and strong memory CD4+ and CD8+ T cells induced by SARS-CoV-2 in UK convalescent individuals following COVID-19. Nat Immunol (2020) 12:1336–45. 10.1101/2020.06.05.134551 PMC7611020 32887977

[50] Giménez E Albert E Torres I Remigia MJ Alcaraz MJ Galindo MJ SARS-CoV-2-reactive interferon-γ-producing CD8+ T cells in patients hospitalized with coronavirus disease 2019. J Med Virol (2020) 1–8. 10.1002/jmv.26213 32579268 PMC7361624

[51] Zheng H-Y Zhang M Yang C-X Zhang N Wang X-C Yang X-P Elevated exhaustion levels and reduced functional diversity of T cells in peripheral blood may predict severe progression in COVID-19 patients. Cell Mol Immunol (2020) 175:541–3. 10.1038/s41423-020-0401-3 PMC7091621 32203186

[52] Braun J Loyal L Frentsch M Wendisch D Georg P Kurth F SARS-CoV-2-reactive T cells in healthy donors and patients with COVID-19. Nature (2020) 587:270–4. 10.1101/2020.04.17.20061440 32726801

[53] Bodewes R Fraaij PLA Geelhoed-Mieras MM van Baalen CA Tiddens HAWM van Rossum AMC Annual Vaccination against Influenza Virus Hampers Development of Virus-Specific CD8+ T Cell Immunity in Children. J Virol (2011) 85:11995–2000. 10.1128/jvi.05213-11 PMC3209321 21880755

[54] Mohn KG-I Brokstad KA Islam S Oftung F Tøndel C Aarstad HJ Early Induction of Cross-Reactive CD8+ T-Cell Responses in Tonsils After Live-Attenuated Influenza Vaccination in Children. J Infect Dis (2020) 221:1528–37. 10.1093/infdis/jiz583 PMC7137893 32255493

[55] Baitsch L Baumgaertner P Devêvre E Raghav SK Legat A Barba L Exhaustion of tumor-specific CD8^+^ T cells in metastases from melanoma patients. J Clin Invest (2011) 121:2350–60. 10.1172/jci46102 PMC3104769 21555851

[56] Li J Arévalo MT Chen Y Chen S Zeng M T-cell-mediated cross-strain protective immunity elicited by prime-boost vaccination with a live attenuated influenza vaccine. Int J Infect Dis Ijid Off Publ Int Soc Infect Dis (2014) 27:37–43. 10.1016/j.ijid.2014.05.016 PMC4197066 25172265

[57] Zhang C Maruggi G Shan H Li J Advances in mRNA Vaccines for Infectious Diseases. Front Immunol (2019) 10:594. 10.3389/fimmu.2019.00594 30972078 PMC6446947

[58] Ho NI Veld LG Raaijmakers TK Adema GJ Adjuvants Enhancing Cross-Presentation by Dendritic Cells: The Key to More Effective Vaccines? Front Immunol (2018) 9:2874. 10.3389/fimmu.2018.02874 30619259 PMC6300500

[59] Blander MJ The comings and goings of MHC class I molecules herald a new dawn in cross-presentation. Immunol Rev (2016) 272:65–79. 10.1111/imr.12428 27319343 PMC4942502

[60] Montealegre S van Endert P MHC Class I Cross-Presentation: Stage Lights on Sec22b. Trends Immunol (2017) 38:618–21. 10.1016/j.it.2017.07.002 28743621

[61] de Brito C Tomkowiak M Ghittoni R Caux C Leverrier Y Marvel J CpG promotes cross-presentation of dead cell-associated antigens by pre-CD8α+ dendritic cells [corrected]. J Immunol (Baltimore Md: 1950) (2011) 186:1503–11. 10.4049/jimmunol.1001022 21187449

[62] Klechevsky E Flamar A-L Cao Y Blanck J-P Liu M O’Bar A Cross-priming CD8+ T cells by targeting antigens to human dendritic cells through DCIR. Blood (2010) 116:1685–97. 10.1182/blood-2010-01-264960 PMC2947393 20530286

[63] Shen K-Y Song Y-C Chen I-H Leng C-H Chen H-W Li H-J Molecular Mechanisms of TLR2-Mediated Antigen Cross-Presentation in Dendritic Cells. J Immunol (2014) 192:4233–41. 10.4049/jimmunol.1302850 PMC3993050 24683188

[64] Mouriès J Moron G Schlecht G Escriou N Dadaglio G Leclerc C Plasmacytoid dendritic cells efficiently cross-prime naive T cells in vivo after TLR activation. Blood (2008) 112:3713–22. 10.1182/blood-2008-03-146290 PMC2572799 18698004

[65] Schreibelt G Klinkenberg LJ Cruz LJ Tacken PJ Tel J Kreutz M Vries JI de. The C-type lectin receptor CLEC9A mediates antigen uptake and (cross-)presentation by human blood BDCA3+ myeloid dendritic cells. Blood (2012) 119:2284–92. 10.1182/blood-2011-08-373944 22234694

[66] Nair-Gupta P Baccarini A Tung N Seyffer F Florey O Huang Y TLR Signals Induce Phagosomal MHC-I Delivery from the Endosomal Recycling Compartment to Allow Cross-Presentation. Cell (2014) 158:506–21. 10.1016/j.cell.2014.04.054 PMC4212008 25083866

[67] Crespo MI Zacca ER Núñez NG Ranocchia RP Maccioni M Maletto BA TLR7 Triggering with Polyuridylic Acid Promotes Cross-Presentation in CD8α+ Conventional Dendritic Cells by Enhancing Antigen Preservation and MHC Class I Antigen Permanence on the Dendritic Cell Surface. J Immunol (2013) 190:948–60. 10.4049/jimmunol.1102725 23284054

[68] Alloatti A Kotsias F Pauwels A-M Carpier J-M Jouve M Timmerman E Toll-like Receptor 4 Engagement on Dendritic Cells Restrains Phago-Lysosome Fusion and Promotes Cross-Presentation of Antigens. Immunity (2015) 43:1087–100. 10.1016/j.immuni.2015.11.006 26682983

[69] Montecino-Rodriguez E Berent-Maoz B Dorshkind K Causes, consequences, and reversal of immune system aging. J Clin Invest (2013) 123:958–65. 10.1172/jci64096 PMC3582124 23454758

[70] Min H Montecino-Rodriguez E Dorshkind K Reduction in the Developmental Potential of Intrathymic T Cell Progenitors with Age. J Immunol (2004) 173:245–50. 10.4049/jimmunol.173.1.245 15210781

[71] Heng TSP Goldberg GL Gray DHD Sutherland JS Chidgey AP Boyd RL Effects of Castration on Thymocyte Development in Two Different Models of Thymic Involution. J Immunol (2005) 175:2982–93. 10.4049/jimmunol.175.5.2982 16116185

[72] Palmer DB The Effect of Age on Thymic Function. Front Immunol (2013) 4:316. 10.3389/fimmu.2013.00316 24109481 PMC3791471

[73] Taub DD Longo DL Insights into thymic aging and regeneration. Immunol Rev (2005) 205:72–93. 10.1111/j.0105-2896.2005.00275.x 15882346

[74] Pido-Lopez J Imami N Aspinall R Both age and gender affect thymic output: more recent thymic migrants in females than males as they age. Clin Exp Immunol (2001) 125:409–13. 10.1046/j.1365-2249.2001.01640.x PMC1906152 11531948

[75] Ravkov E Slev P Heikal N Thymic output: Assessment of CD4 + recent thymic emigrants and T-Cell receptor excision circles in infants. Cytom Part B Clin Cytom (2016) 92:249–57. 10.1002/cyto.b.21341 26566232

[76] Schatorjé EJH Gemen EFA Driessen GJA Leuvenink J van Hout RWNM de Vries E Paediatric Reference Values for the Peripheral T cell Compartment. Scand J Immunol (2012) 75:436–44. 10.1111/j.1365-3083.2012.02671.x 22420532

[77] Goronzy JJ Fang F Cavanagh MM Qi Q Weyand CM Naive T Cell Maintenance and Function in Human Aging. J Immunol (2015) 194:4073–80. 10.4049/jimmunol.1500046 PMC4452284 25888703

[78] Salam N Rane S Das R Faulkner M Kandpal RG T cell ageing: Effects of age on development, survival & function. Indian J Med Res (2013) 138(5):595–608. 24434315 PMC3928693

[79] Schönland SO Zimmer JK Lopez-Benitez CM Widmann T Ramin KD Goronzy JJ Homeostatic control of T-cell generation in neonates. Blood (2003) 102:1428–34. 10.1182/blood-2002-11-3591 12714521

[80] Deacock SJ Schwarer AP Bridge J Batchelor JR Goldman JM Lechler RI Evidence that umbilical cord blood contains a higher frequency of HLA class II-specific alloreactive T cells than adult peripheral blood. Transplantation (1992) 53:1128–34. 10.1097/00007890-199205000-00028 1374946

[81] Thome JJC Bickham KL Ohmura Y Kubota M Matsuoka N Gordon C Early-life compartmentalization of human T cell differentiation and regulatory function in mucosal and lymphoid tissues. Nat Med (2016) 22:72–7. 10.1038/nm.4008 PMC4703455 26657141

[82] Crispín JC Tsokos GC Human TCR-alpha beta+ CD4- CD8- T cells can derive from CD8+ T cells and display an inflammatory effector phenotype. J Immunol Baltim Md 1950 (2009) 183:4675–81. 10.4049/jimmunol.0901533 PMC2878279 19734235

[83] Lewis DB Wilson CB Infectious Diseases of the Fetus and Newborn. Sect Gen Inf (2011) 80–191. 10.1016/b978-1-4160-6400-8.00004-3

[84] Sato K Kawasaki H Nagayama H Enomoto M Morimoto C Tadokoro K Chemokine Receptor Expressions and Responsiveness of Cord Blood T Cells. J Immunol (2001) 166:1659–66. 10.4049/jimmunol.166.3.1659 11160208

[85] Zdolsek HA Ernerudh J Holt PG Nilsson J Björkstén B Expression of the T–Cell Markers CD3, CD4 and CD8 in Healthy and Atopic Children during the First 18 Months of Life. Int Arch Allergy Imm (1999) 119:6–12. 10.1159/000024169 10341315

[86] Azzam HS Grinberg A Lui K Shen H Shores EW Love PE Expression Is Developmentally Regulated By T Cell Receptor (TCR) Signals and TCR Avidity. J Exp Med (1998) 188:2301–11. 10.1084/jem.188.12.2301 PMC2212429 9858516

[87] Weng N-P Akbar AN Goronzy J CD28(-) T cells: their role in the age-associated decline of immune function. Trends Immunol (2009) 30:306–12. 10.1016/j.it.2009.03.013 PMC2801888 19540809

[88] Sherman GG Scott LE Galpin JS Kuhn L Tiemessen CT Simmank K CD38 Expression on CD8+ T Cells as a Prognostic Marker in Vertically HIV-Infected Pediatric Patients. Pediatr Res (2002) 51:740–5. 10.1203/00006450-200206000-00013 12032270

[89] Anfossi N Pascal V Vivier E Ugolini S Biology of T memory type 1 cells. Immunol Rev (2001) 181:269–78. 10.1034/j.1600-065x.2001.1810123.x 11513148

[90] Clénet M-L Gagnon F Moratalla AC Viel EC Arbour N Peripheral human CD4+CD8+ T lymphocytes exhibit a memory phenotype and enhanced responses to IL-2, IL-7 and IL-15. Sci Rep-uk (2017) 7:11612. 10.1038/s41598-017-11926-2 PMC5599513 28912605

[91] Zenarruzabeitia O Vitallé J García-Obregón S Astigarraga I Eguizabal C Santos S The expression and function of human CD300 receptors on blood circulating mononuclear cells are distinct in neonates and adults. Sci Rep-uk (2016) 6:32693. 10.1038/srep32693 PMC5011699 27595670

[92] Fagnoni FF Vescovini R Mazzola M Bologna G Nigro E Lavagetto G Expansion of cytotoxic CD8 + CD28 – T cells in healthy ageing people, including centenarians. Immunology (1996) 88:501–7. 10.1046/j.1365-2567.1996.d01-689.x PMC1456634 8881749

[93] Glaría E Valledor AF Roles of CD38 in the Immune Response to Infection. Cells (2020) 9:228. 10.3390/cells9010228 PMC7017097 31963337

[94] Martino MD Rossi ME Azzari C Gelli MG Galli L Vierucci A Different Meaning of CD38 Molecule Expression on CD4+ and CD8+ Cells of Children Perinatally Infected with Human Immunodeficiency Virus Type 1 Infection Surviving Longer than Five Years. Pediatr Res (1998) 43:752–8. 10.1203/00006450-199806000-00007 9621984

[95] Schlesinger M Peters V Jiang JD Roboz JP Bekesi JG Increased Expression of Activation Markers on CD8 Lymphocytes in Children with Human Immunodeficiency Virus-1 Infection. Pediatr Res (1995) 38:390–6. 10.1203/00006450-199509000-00020 7494665

[96] Rudolph ME McArthur MA Barnes RS Magder LS Chen WH Sztein MB Differences Between Pediatric and Adult T Cell Responses to In Vitro Staphylococcal Enterotoxin B Stimulation. Front Immunol (2018) 9:498. 10.3389/fimmu.2018.00498 29616025 PMC5869216

[97] Warren HS Rana PM Rieger DT Hewitt KA Dahlstrom JE Kent AL CD8 T cells expressing killer Ig-like receptors and NKG2A are present in cord blood and express a more naïve phenotype than their counterparts in adult blood. J Leukocyte Biol (2006) 79:1252–9. 10.1189/jlb.0905536 16574769

[98] Björkström NK Béziat V Cichocki F Liu LL Levine J Larsson S CD8 T cells express randomly selected KIRs with distinct specificities compared with NK cells. Blood (2012) 120:3455–65. 10.1182/blood-2012-03-416867 PMC3482857 22968455

[99] Basatena N-KS al MacNamara A Vine AM Thio CL Astemborski J Usuku K KIR2DL2 Enhances Protective and Detrimental HLA Class I-Mediated Immunity in Chronic Viral Infection. PloS Pathog (2011) 7:e1002270. 10.1371/journal.ppat.1002270 22022261 PMC3192839

[100] Vitallé J Terrén I Orrantia A Zenarruzabeitia O Borrego F CD300 receptor family in viral infections. Eur J Immunol (2019) 49:364–74. 10.1002/eji.201847951 30485414

[101] Rudd BD Neonatal T Cells: A Reinterpretation. Annu Rev Immunol (2020) 38:229–47. 10.1146/annurev-immunol-091319-083608 PMC7369171 31928469

[102] Manolios N Ali M Bender V T-cell antigen receptor (TCR) transmembrane peptides: A new paradigm for the treatment of autoimmune diseases. Cell Adhes Migr (2010) 4:273–83. 10.4161/cam.4.2.11909 PMC2900625 20431344

[103] Venturi V Nzingha K Amos TG Charles WC Dekhtiarenko I Cicin-Sain L The Neonatal CD8+ T Cell Repertoire Rapidly Diversifies during Persistent Viral Infection. J Immunol (2016) 196:1604–16. 10.4049/jimmunol.1501867 PMC4744528 26764033

[104] Haynes L Maue AC Effects of aging on T cell function. Curr Opin Immunol (2009) 21:414–7. 10.1016/j.coi.2009.05.009 PMC3800142 19500967

[105] Philbin VJ Dowling DJ Gallington LC Cortés G Tan Z Suter EE Imidazoquinoline Toll-like receptor 8 agonists activate human newborn monocytes and dendritic cells through adenosine-refractory and caspase-1-dependent pathways. J Allergy Clin Immunol (2012) 130:195–204.e9. 10.1016/j.jaci.2012.02.042 22521247 PMC3387351

[106] Nelson MH Chu C Milligan GN Effector function and efficacy of CD8+ T cells activated in the absence of IFN-γ. FASEB J (2008) 22:7–855. 10.1096/fasebj.22.1_supplement.855.7

[107] Vukmanovic-Stejic M Vyas B Gorak-Stolinska P Noble A Kemeny DM Human Tc1 and Tc2/Tc0 CD8 T-cell clones display distinct cell surface and functional phenotypes. Blood (2000) 95:231–40. 10.1182/blood.V95.1.231 10607707

[108] Siefker DT Vu L You D McBride A Taylor R Jones TL Respiratory Syncytial Virus Disease Severity is Associated with Distinct CD8+ T Cell Profiles. Am J Resp Crit Care (2019) 201(3):325–34. 10.1164/rccm.201903-0588oc PMC6999109 31644878

[109] Makris D Lazarou S Alexandrakis M Kourelis TV Tzanakis N Kyriakou D Tc2 response at the onset of COPD exacerbations. Chest (2008) 134:483–8. 10.1378/chest.07-2626 18490406

[110] Kollmann TR Crabtree J Rein-Weston A Blimkie D Thommai F Wang XY Neonatal Innate TLR-Mediated Responses Are Distinct from Those of Adults. J Immunol (2009) 183:7150–60. 10.4049/jimmunol.0901481 PMC4556237 19917677

[111] Vitallé J Terrén I Gamboa-Urquijo L Orrantia A Tarancón-Díez L Genebat M Polyfunctional HIV-1 specific response by CD8+ T lymphocytes expressing high levels of CD300a. Sci Rep-uk (2020) 10:6070. 10.1038/s41598-020-63025-4 PMC7142067 32269232

[112] Galindo-Albarrán AO López-Portales OH Gutiérrez-Reyna DY Rodríguez-Jorge O Sánchez-Villanueva JA Ramírez-Pliego O CD8+ T Cells from Human Neonates Are Biased toward an Innate Immune Response. Cell Rep (2016) 17:2151–60. 10.1016/j.celrep.2016.10.056 27851975

[113] Lee Y-C Lin S-J Neonatal Natural Killer Cell Function: Relevance to Antiviral Immune Defense. Clin Dev Immunol (2013) 2013:1–6. 10.1155/2013/427696 PMC3770027 24066005

[114] Nikolich-Žugich J Aging of the T Cell Compartment in Mice and Humans: From No Naive Expectations to Foggy Memories. J Immunol (2014) 193:2622–9. 10.4049/jimmunol.1401174 PMC4157314 25193936

[115] Chen G Lustig A Weng N T Cell Aging: A Review of the Transcriptional Changes Determined from Genome-Wide Analysis. Front Immunol (2013) 4:121. 10.3389/fimmu.2013.00121 23730304 PMC3657702

[116] Smith NL Wissink E Wang J Pinello JF Davenport MP Grimson A Rapid Proliferation and Differentiation Impairs the Development of Memory CD8+ T Cells in Early Life. J Immunol (2014) 193:177–84. 10.4049/jimmunol.1400553 PMC4065808 24850719

[117] Wissink EM Smith NL Spektor R Rudd BD Grimson A MicroRNAs and Their Targets Are Differentially Regulated in Adult and Neonatal Mouse CD8+ T Cells. Genetics (2015) 201:1017–30. 10.1534/genetics.115.179176 PMC4649632 26416483

[118] Lundström W Fewkes NM Mackall CL IL-7 in human health and disease. Semin Immunol (2012) 24:218–24. 10.1016/j.smim.2012.02.005 PMC3358500 22410365

[119] Capitini CM Chisti AA Mackall CL Modulating T cell Homeostasis with IL-7: Preclinical and Clinical Studies. J Intern Med (2009) 266:141–53. 10.1111/j.1365-2796.2009.02085.x PMC2797310 19623690

[120] Yan F Mo X Liu J Ye S Zeng X Chen D Thymic function in the regulation of T cells, and molecular mechanisms underlying the modulation of cytokines and stress signaling. Mol Med Rep (2017) 16:7175–84. 10.3892/mmr.2017.7525 PMC5865843 28944829

[121] Nikolich-Žugich J T cell aging. J Exp Med (2005) 201:837–40. 10.1084/jem.20050341 PMC2213096 15781575

[122] Kazi JU Rönnstrand L FMS-like Tyrosine Kinase 3/FLT3: From Basic Science to Clinical Implications. Physiol Rev (2019) 99:1433–66. 10.1152/physrev.00029.2018 31066629

[123] Dong J McPherson CM Stambrook PJ Flt-3 Ligand: A Potent Dendritic Cell Stimulator and Novel Antitumor. Cancer Biol Ther (2002) 1:486–9. 10.4161/cbt.1.5.161 12496473

[124] Choi Y Chang J Viral vectors for vaccine applications. Clin Exp Vaccine Res (2013) 2:97–105. 10.7774/cevr.2013.2.2.97 23858400 PMC3710930

[125] Truckenmiller ME Norbury CC Viral vectors for inducing CD8+T cell responses. Expert Opin Biol Th (2004) 4:861–8. 10.1517/14712598.4.6.861 15174968

[126] Saade F Petrovsky N Technologies for enhanced efficacy of DNA vaccines. Expert Rev Vaccines (2012) 11:189–209. 10.1586/erv.11.188 22309668 PMC3293989

[127] Sabbaghi A Ghaemi A Molecular Adjuvants for DNA Vaccines: Application, Design, Preparation, and Formulation. Methods Mol Biol Clifton N J (2021) 2197:87–112. 10.1007/978-1-0716-0872-2_5 32827133

[128] Levy O Goriely S Kollmann TR Immune response to vaccine adjuvants during the first year of life. Vaccine (2013) 31:2500–5. 10.1016/j.vaccine.2012.10.016 PMC4048858 23085363

[129] van den Biggelaar A Pomat W Bosco A Phuanukoonnon S Devitt CJ Nadal-Sims MA Pneumococcal conjugate vaccination at birth in a high-risk setting: No evidence for neonatal T-cell tolerance. Vaccine (2011) 29:5414–20. 10.1016/j.vaccine.2011.05.065 PMC3146700 21645573

[130] Borriello F Pietrasanta C Lai JCYC Walsh LM Sharma P O’Driscoll DN Identification and Characterization of Stimulator of Interferon Genes As a Robust Adjuvant Target for Early Life Immunization. Front Immunol (2017) 8:1772. 10.3389/fimmu.2017.01772 29312305 PMC5732947

[131] Bevan MJ Cross-priming for a secondary cytotoxic response to minor H antigens with H-2 congenial cells which do not cross-react in the cytotoxic assay. J Immunol (1976) 117:2233–8. 10.1084/jem.143.5.1283 20660360

[132] Bevan MJ Minor H Antigens Introduced on H-2 Different Stimulating Cells Cross-React at the Cytotoxic T Cell Level during in Vivo Priming. J Immunol (1976) 117:2233–8. 825578

[133] Bachem A Güttler S Hartung E Ebstein F Schaefer M Tannert A Superior antigen cross-presentation and XCR1 expression define human CD11c+CD141+ cells as homologues of mouse CD8+ dendritic cellsHuman CD141+ DCs correspond to mouse CD8+ DCs. J Exp Med (2010) 207:1273–81. 10.1084/jem.20100348 PMC2882837 20479115

[134] Haniffa M Gunawan M Jardine L Human skin dendritic cells in health and disease. J Dermatol Sci (2014) 77:85–92. 10.1016/j.jdermsci.2014.08.012 25301671 PMC4728191

[135] Robbins SH Walzer T Dembélé D Thibault C Defays A Bessou G Novel insights into the relationships between dendritic cell subsets in human and mouse revealed by genome-wide expression profiling. Genome Biol (2008) 9:R17. 10.1186/gb-2008-9-1-r17 18218067 PMC2395256

[136] Crozat K Guiton R Contreras V Feuillet V Dutertre C-A Ventre E The XC chemokine receptor 1 is a conserved selective marker of mammalian cells homologous to mouse CD8alpha+ dendritic cells. J Exp Med (2010) 207:1283–92. 10.1084/jem.20100223 PMC2882835 20479118

[137] Yu CI Becker C Metang P Marches F Wang Y Toshiyuki H Human CD141+ Dendritic Cells Induce CD4+ T Cells To Produce Type 2 Cytokines. J Immunol (2014) 193:4335–43. 10.4049/jimmunol.1401159 PMC4201960 25246496

[138] Segura E Valladeau-Guilemond J Donnadieu M-H Sastre-Garau X Soumelis V Amigorena S Characterization of resident and migratory dendritic cells in human lymph nodes. J Exp Med (2012) 209:653–60. 10.1084/jem.20111457 PMC3328358 22430490

[139] Flinsenberg TWH Compeer EB Koning D Klein M Amelung FJ van Baarle D Fcγ receptor antigen targeting potentiates cross-presentation by human blood and lymphoid tissue BDCA-3+ dendritic cells. Blood (2012) 120:5163–72. 10.1182/blood-2012-06-434498 23093620

[140] Mittag D Proietto AI Loudovaris T Mannering SI Vremec D Shortman K Human Dendritic Cell Subsets from Spleen and Blood Are Similar in Phenotype and Function but Modified by Donor Health Status. J Immunol (2011) 186:6207–17. 10.4049/jimmunol.1002632 21515786

[141] Rojas IML Mok W-H Pearson FE Minoda Y Kenna TJ Barnard RT Human Blood CD1c+ Dendritic Cells Promote Th1 and Th17 Effector Function in Memory CD4+ T Cells. Front Immunol (2017) 8:971. 10.3389/fimmu.2017.00971 28878767 PMC5572390

[142] Jongbloed SL Kassianos AJ McDonald KJ Clark GJ Ju X Angel CE Human CD141+ (BDCA-3)+ dendritic cells (DCs) represent a unique myeloid DC subset that cross-presents necrotic cell antigens. J Exp Med (2010) 207:1247–60. 10.1084/jem.20092140 PMC2882828 20479116

[143] Igyártó BZ Haley K Ortner D Bobr A Gerami-Nejad M Edelson BT Skin-Resident Murine Dendritic Cell Subsets Promote Distinct and Opposing Antigen-Specific T Helper Cell Responses. Immunity (2011) 35:260–72. 10.1016/j.immuni.2011.06.005 PMC3163010 21782478

[144] Segura E Durand M Amigorena S Similar antigen cross-presentation capacity and phagocytic functions in all freshly isolated human lymphoid organ–resident dendritic cells. JEM (nd) (210) 210 (10):1035–47. 10.1084/jem.20121103 PMC3646495 23569327

[145] Nierkens S Tel J Janssen E Adema GJ Antigen cross-presentation by dendritic cell subsets: one general or all sergeants? Trends Immunol (2013) 34:361–70. 10.1016/j.it.2013.02.007 PMC4351710 23540650

[146] Hoeffel G Ripoche A-C Matheoud D Nascimbeni M Escriou N Lebon P Antigen Crosspresentation by Human Plasmacytoid Dendritic Cells. Immunity (2007) 27:481–92. 10.1016/j.immuni.2007.07.021 17869134

[147] Solano-Gálvez SG Tovar-Torres SM Tron-Gómez MS Weiser-Smeke AE Álvarez-Hernández DA Franyuti-Kelly GA Human Dendritic Cells: Ontogeny and Their Subsets in Health and Disease. Med Sci (2018) 6:88. 10.3390/medsci6040088 PMC6313400 30297662

[148] Swiecki M Colonna M The multifaceted biology of plasmacytoid dendritic cells. Nat Rev Immunol (2015) 15:471–85. 10.1038/nri3865 PMC4808588 26160613

[149] Novak N Bieber T The role of dendritic cell subtypes in the pathophysiology of atopic dermatitis. J Am Acad Dermatol (2005) 53:S171–6. 10.1016/j.jaad.2005.04.060 16021172

[150] Gutiérrez-Martínez E Planès R Anselmi G Reynolds M Menezes S Adiko AC Cross-Presentation of Cell-Associated Antigens by MHC Class I in Dendritic Cell Subsets. Front Immunol (2015) 6:363. 10.3389/fimmu.2015.00363 26236315 PMC4505393

[151] Klechevsky E Morita R Liu M Cao Y Coquery S Thompson-Snipes L Functional Specializations of Human Epidermal Langerhans Cells and CD14+ Dermal Dendritic Cells. Immunity (2008) 29:497–510. 10.1016/j.immuni.2008.07.013 18789730 PMC2688399

[152] Chu C-C Ali N Karagiannis P Meglio PD Skowera A Napolitano L Resident CD141 (BDCA3)+ dendritic cells in human skin produce IL-10 and induce regulatory T cells that suppress skin inflammation. J Exp Med (2012) 209:935–45. 10.1084/jem.20112583 PMC3348099 22547651

[153] Penel-Sotirakis K Simonazzi E Péguet-Navarro J Rozières A Differential Capacity of Human Skin Dendritic Cells to Polarize CD4+T Cells into IL-17, IL-21 and IL-22 Producing Cells. PloS One (2012) 7:e45680. 10.1371/journal.pone.0045680 23226194 PMC3511471

[154] Collin M McGovern N Haniffa M Human dendritic cell subsets. Immunology (2013) 140:22–30. 10.1111/imm.12117 23621371 PMC3809702

[155] Hänsel A Günther C Ingwersen J Starke J Schmitz M Bachmann M Human slan (6-sulfo LacNAc) dendritic cells are inflammatory dermal dendritic cells in psoriasis and drive strong Th17/Th1 T-cell responses. J Allergy Clin Immun (2011) 127:787–794.e9. 10.1016/j.jaci.2010.12.009 21377044

[156] Micheletti A Finotti G Calzetti F Lonardi S Zoratti E Bugatti M slan/M-DC8+ cells constitute a distinct subset of dendritic cells in human tonsils. Oncotarget (2016) 7:161–75. 10.18632/oncotarget.6660 PMC4807990 26695549

[157] Tezuka H Abe Y Iwata M Takeuchi H Ishikawa H Matsushita M Regulation of IgA production by naturally occurring TNF/iNOS-producing dendritic cells. Nature (2007) 448:929–33. 10.1038/nature06033 17713535

[158] Zaba LC Krueger JG Lowes MA Resident and “Inflammatory” Dendritic Cells in Human Skin. J Invest Dermatol (2009) 129:302–8. 10.1038/jid.2008.225 PMC2746703 18685620

[159] Schmid M Wege AK Ritter U Characteristics of “Tip-DCs and MDSCs” and Their Potential Role in Leishmaniasis. Front Microbiol (2012) 3:74. 10.3389/fmicb.2012.00074 22416241 PMC3298847

[160] McGovern N Chan JKY Ginhoux F Dendritic cells in humans—from fetus to adult. Int Immunol (2015) 27:65–72. 10.1093/intimm/dxu091 25323843

[161] Collin M Bigley V Human dendritic cell subsets: an update. Immunology (2018) 154:3–20. 10.1111/imm.12888 29313948 PMC5904714

[162] Audsley KM McDonnell AM Waithman J Cross-Presenting XCR1+ Dendritic Cells as Targets for Cancer Immunotherapy. Cells (2020) 9:565. 10.3390/cells9030565 PMC7140519 32121071

[163] Fu C Peng P Loschko J Feng L Pham P Cui W Plasmacytoid dendritic cells cross-prime naive CD8 T cells by transferring antigen to conventional dendritic cells through exosomes. Proc Natl Acad Sci (2020) 117:23730–41. 10.1073/pnas.2002345117 PMC7519282 32879009

[164] Schnurr M Chen Q Shin A Chen W Toy T Jenderek C Tumor antigen processing and presentation depend critically on dendritic cell type and the mode of antigen delivery. Blood (2005) 105:2465–72. 10.1182/blood-2004-08-3105 15546948

[165] Schüller SS Sadeghi K Wisgrill L Dangl A Diesner SC Prusa AR Preterm neonates display altered plasmacytoid dendritic cell function and morphology. J Leukocyte Biol (2013) 93:781–8. 10.1189/jlb.1011525 23401600

[166] Zhang X Lepelley A Azria E Lebon P Roguet G Schwartz O Neonatal Plasmacytoid Dendritic Cells (pDCs) Display Subset Variation but Can Elicit Potent Anti-Viral Innate Responses. PloS One (2013) 8:e52003. 10.1371/journal.pone.0052003 23326320 PMC3542339

[167] Nunes RD Zandavalli FM Association between maternal and fetal factors and quality of cord blood as a source of stem cells. Rev Bras Hematol E Hemoterapia (2014) 37:38–42. 10.1016/j.bjhh.2014.07.023 PMC4318845 25638766

[168] Savina A Peres A Cebrian I Carmo N Moita C Hacohen N The Small GTPase Rac2 Controls Phagosomal Alkalinization and Antigen Crosspresentation Selectively in CD8+ Dendritic Cells. Immunity (2009) 30:544–55. 10.1016/j.immuni.2009.01.013 19328020

[169] Jancic C Savina A Wasmeier C Tolmachova T El-Benna J Dang PM-C Rab27a regulates phagosomal pH and NADPH oxidase recruitment to dendritic cell phagosomes. Nat Cell Biol (2007) 9:367–78. 10.1038/ncb1552 17351642

[170] Mantegazza AR Savina A Vermeulen M Pérez L Geffner J Hermine O NADPH oxidase controls phagosomal pH and antigen cross-presentation in human dendritic cells. Blood (2008) 112:4712–22. 10.1182/blood-2008-01-134791 PMC2597138 18682599

[171] Villadangos JA Ploegh HL Proteolysis in MHC Class II Antigen Presentation. Immunity (2000) 12:233–9. 10.1016/s1074-7613(00)80176-4 10755610

[172] Burgdorf S Lukacs-Kornek V Kurts C The Mannose Receptor Mediates Uptake of Soluble but Not of Cell-Associated Antigen for Cross-Presentation. J Immunol (2006) 176:6770–6. 10.4049/jimmunol.176.11.6770 16709836

[173] Roy R Touaibia M Application of Multivalent Mannosylated Dendrimers in Glycobiology. Elsevier (2007) 821–70. 10.1016/b978-044451967-2/00112-4

[174] Idoyaga J Lubkin A Fiorese C Lahoud MH Caminschi I Huang Y Comparable T helper 1 (Th1) and CD8 T-cell immunity by targeting HIV gag p24 to CD8 dendritic cells within antibodies to Langerin, DEC205, and Clec9A. Proc Natl Acad Sci (2011) 108:2384–9. 10.1073/pnas.1019547108 PMC3038758 21262813

[175] Tacken PJ Ginter W Berod L Cruz LJ Joosten B Sparwasser T Targeting DC-SIGN via its neck region leads to prolonged antigen residence in early endosomes, delayed lysosomal degradation, and cross-presentation. Blood (2011) 118:4111–9. 10.1182/blood-2011-04-346957 21860028

[176] Murshid A Borges TJ Bonorino C Lang BJ Calderwood SK Immunological Outcomes Mediated Upon Binding of Heat Shock Proteins to Scavenger Receptors SCARF1 and LOX-1, and Endocytosis by Mononuclear Phagocytes. Front Immunol (2020) 10:3035. 10.3389/fimmu.2019.03035 31998315 PMC6968791

[177] Filias A Theodorou GL Mouzopoulou S Varvarigou AA Karakantza SM Phagocytic ability of neutrophils and monocytes in neonates. BMC Pediatr (2011) 11(29):1–6. 10.1186/1471-2431-11-29 21492472 PMC3094219

[178] Henneke P Berner R Interaction of Neonatal Phagocytes with Group B Streptococcus: Recognition and Response. Infect Immun (2006) 74:3085–95. 10.1128/iai.01551-05 PMC1479263 16714536

[179] Prosser A Hibbert J Strunk T Kok CH Simmer K Richmond P Phagocytosis of neonatal pathogens by peripheral blood neutrophils and monocytes from newborn preterm and term infants. Pediatr Res (2013) 74:503–10. 10.1038/pr.2013.145 23999070

[180] Gros M Amigorena S Regulation of Antigen Export to the Cytosol During Cross-Presentation. Front Immunol (2019) 10:41. 10.3389/fimmu.2019.00041 30745902 PMC6360170

[181] Li B Lijuan H Cross-presentation of Exogenous Antigens. Transfus Clin Biol (2019) 26:346–51. 10.1016/j.tracli.2019.01.006 30797678

[182] Ménager J Ebstein F Oger R Hulin P Nedellec S Duverger E Cross-Presentation of Synthetic Long Peptides by Human Dendritic Cells: A Process Dependent on ERAD Component p97/VCP but Not sec61 and/or Derlin-1. PloS One (2014) 9:e89897. 10.1371/journal.pone.0089897 24587108 PMC3937416

[183] Grotzke JE Kozik P Morel J-D Impens F Pietrosemoli N Cresswell P Sec61 blockade by mycolactone inhibits antigen cross-presentation independently of endosome-to-cytosol export. Proc Natl Acad Sci (2017) 114:E5910–9. 10.1073/pnas.1705242114 PMC5530691 28679634

[184] Blander JM Regulation of the Cell Biology of Antigen Cross Presentation. Annu Rev Immunolo (2018) 10AD) 36:27.1–27.37. 10.1146/annurev-immunol-041015-055523 PMC6430635 29490164

[185] Hämälistö S Stahl JL Favaro E Yang Q Liu B Christoffersen L Spatially and temporally defined lysosomal leakage facilitates mitotic chromosome segregation. Nat Commun (2020) 11:229. 10.1038/s41467-019-14009-0 31932607 PMC6957743

[186] Wu SJ Niknafs YS Kim SH Oravecz-Wilson K Zajac C Toubai T A Critical Analysis of the Role of SNARE Protein SEC22B in Antigen Cross-Presentation. Cell Rep (2017) 19:2645–56. 10.1016/j.celrep.2017.06.013 PMC5539946 28658614

[187] Montealegre S Endert P van. MHC Class I Cross-Presentation: Stage Lights on Sec22b. Trends Immunol (2017) 38:618–21. 10.1016/j.it.2017.07.002 28743621

[188] Embgenbroich M Burgdorf S Current Concepts of Antigen Cross-Presentation. Front Immunol (2018) 9:1643. 10.3389/fimmu.2018.01643 30061897 PMC6054923

[189] Cebrian I Visentin G Blanchard N Jouve M Bobard A Moita C Sec22b Regulates Phagosomal Maturation and Antigen Crosspresentation by Dendritic Cells. Cell (2011) 147:1355–68. 10.1016/j.cell.2011.11.021 22153078

[190] Burgdorf S Schölz C Kautz A Tampé R Kurts C Spatial and mechanistic separation of cross-presentation and endogenous antigen presentation. Nat Immunol (2008) 9:558–66. 10.1038/ni.1601 18376402

[191] Merzougui N Kratzer R Saveanu L Endert P van. A proteasome-dependent, TAP-independent pathway for cross-presentation of phagocytosed antigen. EMBO Rep (2011) 12:1257–64. 10.1038/embor.2011.203 PMC3245693 22037009

[192] Vigneron N Ferrari V den Eynde BJV Cresswell P Leonhardt RM Cytosolic Processing Governs TAP-Independent Presentation of a Critical Melanoma Antigen. J Immunol (2018) 201:1875–88. 10.4049/jimmunol.1701479 PMC6457910 30135181

[193] Marijt KA van Hall T To TAP or not to TAP: alternative peptides for immunotherapy of cancer. Curr Opin Immunol (2020) 64:15–9. 10.1016/j.coi.2019.12.004 31952027

[194] Alloatti A Kotsias F Magalhaes J Amigorena S Dendritic cell maturation and cross-presentation: timing matters! Immunol Rev (2016) 272:97–108. 10.1111/imr.12432 27319345 PMC6680313

[195] Kollmann TR Way SS Harowicz HL Hajjar AM Wilson CB Deficient MHC class I cross-presentation of soluble antigen by murine neonatal dendritic cells. Blood (2004) 103:4240–2. 10.1182/blood-2003-11-3805 14982880

[196] Kovacsovics-Bankowski M Rock K A phagosome-to-cytosol pathway for exogenous antigens presented on MHC class I molecules. Science (1995) 267:243–6. 10.1126/science.7809629 7809629

[197] Ma W Stroobant V Heirman C Sun Z Thielemans K Mulder A The Vacuolar Pathway of Long Peptide Cross-Presentation Can Be TAP Dependent. J Immunol (2018) 202:451–9. 10.4049/jimmunol.1800353 30559321

[198] Sengupta D Graham M Liu X Cresswell P Proteasomal degradation within endocytic organelles mediates antigen cross-presentation. EMBO J (2019) 38:e99266. 10.15252/embj.201899266 31271236 PMC6694219

[199] Lin M Zhan Y Villadangos JA Lew AM The cell biology of cross-presentation and the role of dendritic cell subsets. Immunol Cell Biol (2008) 86:353–62. 10.1038/icb.2008.3 18268517

[200] Lázaro S Gamarra D Val MD Proteolytic enzymes involved in MHC class I antigen processing: A guerrilla army that partners with the proteasome. Mol Immunol (2015) 68:72–6. 10.1016/j.molimm.2015.04.014 26006050

[201] Velilla PA Rugeles MT Choughnet CA Defective antigen-presenting cell function in human neonates. Clin Immunol (2006) 121:251–9. 10.1016/j.clim.2006.08.010 PMC1764492 17010668

[202] Snapper CM Distinct Immunologic Properties of Soluble Versus Particulate Antigens. Front Immunol (2018) 9:598. 10.3389/fimmu.2018.00598 29619034 PMC5871672

[203] Petersen A Honarvar A Zetterberg M Changes in Activity and Kinetic Properties of the Proteasome in Different Rat Organs during Development and Maturation. Curr Gerontol Geriatr Res (2010) 2010:230697. 10.1155/2010/230697 PMC2850129 20379353

[204] Saez I Vilchez D The Mechanistic Links Between Proteasome Activity, Aging and Agerelated Diseases. Curr Genomics (2014) 15:38–51. 10.2174/138920291501140306113344 24653662 PMC3958958

[205] Welsh RM Bahl K Marshall HD Urban SL Type 1 interferons and antiviral CD8 T-cell responses. PloS Pathog (2012) 8:e1002352. 10.1371/journal.ppat.1002352 22241987 PMC3252364

[206] Kolumam GA Thomas S Thompson LJ Sprent J Murali-Krishna K Type I interferons act directly on CD8 T cells to allow clonal expansion and memory formation in response to viral infection. J Exp Med (2005) 202:637–50. 10.1084/jem.20050821 PMC2212878 16129706

[207] Marr N Wang T-I Kam SHY Hu YS Sharma AA Lam A Attenuation of Respiratory Syncytial Virus–Induced and RIG-I–Dependent Type I IFN Responses in Human Neonates and Very Young Children. J Immunol (2014) 192:948–57. 10.4049/jimmunol.1302007 24391215

[208] Stephens LM Varga SM Function and Modulation of Type I Interferons during Respiratory Syncytial Virus Infection. Nato Adv Sci Inst Se (2020) 8:177. 10.3390/vaccines8020177 PMC7349809 32290326

[209] Upham JW Lee PT Holt BJ Heaton T Prescott SL Sharp MJ Development of Interleukin-12-Producing Capacity throughout Childhood. Infect Immun (2002) 70:6583–8. 10.1128/iai.70.12.6583-6588.2002 PMC133015 12438328

[210] Gutiérrez-Reyna DY Cedillo-Baños A Kempis-Calanis LA Ramírez-Pliego O Bargier L Puthier D IL-12 Signaling Contributes to the Reprogramming of Neonatal CD8+ T Cells. Front Immunol (2020) 11:1089. 10.3389/fimmu.2020.01089 32582178 PMC7292210

[211] Morishima N Owaki T Asakawa M Kamiya S Mizuguchi J Yoshimoto T Augmentation of Effector CD8+ T Cell Generation with Enhanced Granzyme B Expression by IL-27. J Immunol (2005) 175:1686–93. 10.4049/jimmunol.175.3.1686 16034109

[212] Meyer CU Birkholz J Weins N Doganci A Gehring S Zepp F Dendritic cells change IL-27 production pattern during childhood. BMC Res Notes (2015) 8:232. 10.1186/s13104-015-1182-0 26054397 PMC4467631

[213] Lemoine S Jaron B Tabka S Ettreiki C Deriaud E Zhivaki D Dectin-1 activation unlocks IL12A expression and reveals the TH1 potency of neonatal dendritic cells. J Allergy Clin Immun (2015) 136:1355–1368.e15. 10.1016/j.jaci.2015.02.030 25865351

[214] Belderbos ME Levy O Meyaard L Bont L Plasma-mediated immune suppression: a neonatal perspective. Pediatr Allergy Immunol (2013) 24:102–13. 10.1111/pai.12023 23173652

[215] Belderbos ME Levy O Stalpers F Kimpen JL Meyaard L Bont L Neonatal plasma polarizes TLR4-mediated cytokine responses towards low IL-12p70 and high IL-10 production via distinct factors. PloS One (2012) 7:e33419. 10.1371/journal.pone.0033419 22442690 PMC3307729

[216] Oh D-Y Dowling DJ Ahmed S Choi H Brightman S Bergelson I Adjuvant-induced Human Monocyte Secretome Profiles Reveal Adjuvant- and Age-specific Protein Signatures. Mol Cell Proteomics (2016) 15:1877–94. 10.1074/mcp.M115.055541 PMC5083103 26933193

[217] Wen Y Shi Y Alum: an old dog with new tricks. Emerg Microbes Infec (2019) 5:1–5. 10.1038/emi.2016.40 PMC4820675 27004761

[218] Kool M Soullié T van Nimwegen M Willart MAM Muskens F Jung S Alum adjuvant boosts adaptive immunity by inducing uric acid and activating inflammatory dendritic cells. J Exp Med (2008) 205:869–82. 10.1084/jem.20071087 PMC2292225 18362170

[219] Bruchard M Rebé C Derangère V Togbé D Ryffel B Boidot R The receptor NLRP3 is a transcriptional regulator of TH2 differentiation. Nat Immunol (2015) 16:859–70. 10.1038/ni.3202 26098997

[220] Jiang H Wang Q Li L Zeng Q Li H Gong T Turning the Old Adjuvant from Gel to Nanoparticles to Amplify CD8 + T Cell Responses. Adv Sci (2017) 5:1700426. 10.1002/advs.201700426 PMC5770685 29375970

[221] Marciani DJ Elucidating the Mechanisms of Action of Saponin-Derived Adjuvants. Trends Pharmacol Sci (2018) 39:573–85. 10.1016/j.tips.2018.03.005 29655658

[222] Ragupathi G Gardner JR Livingston PO Gin DY Natural and synthetic saponin adjuvant QS-21 for vaccines against cancer. Expert Rev Vaccines (2011) 10:463–70. 10.1586/erv.11.18 PMC3658151 21506644

[223] Soltysik S Wu J-Y Recchia J Wheeler DA Newman MJ Coughlin RT Structure/function studies of QS-21 adjuvant: assessment of triterpene aldehyde and glucuronic acid roles in adjuvant function. Vaccine (1995) 13:1403–10. 10.1016/0264-410x(95)00077-e 8578817

[224] Collins KC Schlosburg JE Lockner JW Bremer PT Ellis BA Janda KD Lipid tucaresol as an adjuvant for methamphetamine vaccine development. Chem Commun Camb Engl (2014) 50:4079–81. 10.1039/c4cc00682h PMC4033696 24615284

[225] den Brok MH Büll C Wassink M de Graaf AM Wagenaars JA Minderman M Saponin-based adjuvants induce cross-presentation in dendritic cells by intracellular lipid body formation. Nat Commun (2016) 7:13324. 10.1038/ncomms13324 27819292 PMC5103066

[226] Nair P Amsen D Blander JM Co-ordination of Incoming and Outgoing Traffic in Antigen-Presenting Cells by Pattern Recognition Receptors and T Cells. Traffic (2011) 12:1669–76. 10.1111/j.1600-0854.2011.01251.x PMC5800850 21762455

[227] Gil-Torregrosa BC Lennon-Duménil AM Kessler B Guermonprez P Ploegh HL Fruci D Control of cross-presentation during dendritic cell maturation. Eur J Immunol (2004) 34:398–407. 10.1002/eji.200324508 14768044

[228] Kawai T Akira S TLR signaling. Cell Death Differ (2006) 13:816–25. 10.1038/sj.cdd.4401850 16410796

[229] Nair-Gupta P Baccarini A Tung N Seyffer F Florey O Huang Y TLR signals induce phagosomal MHC-I delivery from the endosomal recycling compartment to allow cross-presentation. Cell (2014) 158:506–21. 10.1016/j.cell.2014.04.054 PMC4212008 25083866

[230] Blander JM Medzhitov R Toll-dependent selection of microbial antigens for presentation by dendritic cells. Nature (2006) 440:808–12. 10.1038/nature04596 16489357

[231] Santone M Aprea S Wu TYH Cooke MP Mbow ML Valiante NM A new TLR2 agonist promotes cross-presentation by mouse and human antigen presenting cells. Hum Vacc Immunother (2015) 11:2038–50. 10.1080/21645515.2015.1027467 PMC4635931 26024409

[232] Datta SK Redecke V Prilliman KR Takabayashi K Corr M Tallant T A Subset of Toll-Like Receptor Ligands Induces Cross-presentation by Bone Marrow-Derived Dendritic Cells. J Immunol (2003) 170:4102–10. 10.4049/jimmunol.170.8.4102 12682240

[233] Levy O Suter EE Miller RL Wessels MR Unique efficacy of Toll-like receptor 8 agonists in activating human neonatal antigen-presenting cells. Blood (2006) 108:1284–90. 10.1182/blood-2005-12-4821 PMC1895876 16638933

[234] Levy O Coughlin M Cronstein BN Roy RM Desai A Wessels MR The adenosine system selectively inhibits TLR-mediated TNF-alpha production in the human newborn. J Immunol (Baltimore Md: 1950) (2006) 177:1956–66. 10.4049/jimmunol.177.3.1956 PMC2881468 16849509

[235] Levy O Zarember KA Roy RM Cywes C Godowski PJ Wessels MR Selective impairment of TLR-mediated innate immunity in human newborns: neonatal blood plasma reduces monocyte TNF-alpha induction by bacterial lipopeptides, lipopolysaccharide, and imiquimod, but preserves the response to R-848. J Immunol (Baltimore Md: 1950) (2004) 173:4627–34. 10.4049/jimmunol.173.7.4627 15383597

[236] Angelidou A Conti M-G Diray-Arce J Benn CS Shann F Netea MG Licensed Bacille Calmette-Guérin (BCG) formulations differ markedly in bacterial viability, RNA content and innate immune activation. Vaccine (2020) 38:2229–40. 10.1016/j.vaccine.2019.11.060 PMC7556328 32005538

[237] Angelone DF Wessels MR Coughlin M Suter EE Valentini P Kalish LA Innate immunity of the human newborn is polarized toward a high ratio of IL-6/TNF-alpha production in vitro and in vivo. Pediatr Res (2006) 60:205–9. 10.1203/01.pdr.0000228319.10481.ea 16864705

[238] Kollmann TR Levy O Montgomery RR Goriely S Innate Immune Function by Toll-like Receptors: Distinct Responses in Newborns and the Elderly. Immunity (2012) 37:771–83. 10.1016/j.immuni.2012.10.014 PMC3538030 23159225

[239] Cuenca A Wynn J Moldawer L Levy O Role of Innate Immunity in Neonatal Infection. Am J Perinatol (2013) 30:105–12. 10.1055/s-0032-1333412 PMC3959733 23297181

[240] Zhang J-P Yang Y Levy O Chen C Human Neonatal Peripheral Blood Leukocytes Demonstrate Pathogen-Specific Coordinate Expression of TLR2, TLR4/MD2, and MyD88 During Bacterial Infection In Vivo. Pediatr Res (2010) 68:479–83. 10.1203/pdr.0b013e3181f90810 PMC4108899 20805788

